# Effect of Fluoride on Gut Microbiota: A Systematic Review

**DOI:** 10.1093/nutrit/nuae202

**Published:** 2025-03-10

**Authors:** Momina Yasin, Fatemeh Vida Zohoori, Elizabeth Adjoa Kumah, Murali Subramanian, Paul Dean, Caroline Hayley Orr

**Affiliations:** School of Health and Life Sciences, Teesside University, Middlesbrough TS1 3BX, United Kingdom; National Horizons Centre, Teesside University, Darlington DL1 1HG, United Kingdom; School of Health and Life Sciences, Teesside University, Middlesbrough TS1 3BX, United Kingdom; National Horizons Centre, Teesside University, Darlington DL1 1HG, United Kingdom; Liverpool School of Topical Medicine, Liverpool L3 5QA United Kingdom; Population Health Sciences Institute, Faculty of Medical Sciences, Newcastle University, Newcastle-upon-Tyne NE2 4HH, United Kingdom; School of Health and Life Sciences, Teesside University, Middlesbrough TS1 3BX, United Kingdom; National Horizons Centre, Teesside University, Darlington DL1 1HG, United Kingdom; School of Health and Life Sciences, Teesside University, Middlesbrough TS1 3BX, United Kingdom; National Horizons Centre, Teesside University, Darlington DL1 1HG, United Kingdom

**Keywords:** fluoride, gut microbiota, demineralization, remineralization, humans, animals, short chain fatty acids, ^18^F-FDG (fluorine-18 fluorodeoxyglucose)

## Abstract

**Context:**

Fluoride can prevent dental caries by inhibiting demineralization and promoting remineralization of teeth while affecting the physiology of oral microbiota, thus inhibiting cellular enzymes. However, the effect of systemic fluoride on gut microbiota is unknown.

**Objective:**

To explore the impacts of systemic fluoride on gut microbiota composition and abundance and associated functions such as gene and metabolic regulation.

**Data Sources:**

A systematic database search was conducted of MEDLINE, Web of Science, Scopus, PubMed, CINAHL, and Embase to find articles on studies reporting the effects of fluoride on gut microbiota.

**Data Extraction:**

Forty-nine studies were included (n = 42 in animals, 4 of humans, 3 in vitro studies) after screening for title, abstract, and full text using Covidence to check against eligibility criteria. Data were extracted using Covidence and study quality was assessed using the Mixed Method Appraisal Tool by 2 reviewers independently.

**Data Analysis:**

Two human studies of dental fluorosis and 1 of patients with breast cancer (intestinal fluorine-18 fluorodeoxyglucose uptake) showed significant differences in gut microbial composition, with increased relative abundance of Acidobacteria and Proteobacteria, and decreased abundance of Firmicutes and Bacteroidetes. An ex vivo study of human feces indicated that ≤ 2 mg L^–1^ NaF might boost “health-associated” taxa, but concentrations (≥ 10 mg L^–1^ NaF) could increase the ratio of some unhealthy microbes after 24 hours. The animal studies examined the effects of high fluoride doses in water and diet (50-1200 mg L^–1^ NaF) for long-term (1-6 months) and short-term (6 hours to 7 days) exposure, with all showing a significant disturbance in the Firmicutes to Bacteroidota ratio.

**Conclusion:**

In humans, high doses potentially may be detrimental to the microbiome, whereas ≤ 2 mg L^–1^ NaF had positive effects. Similarly, in animals, ≥ 50 mg L^–1^ NaF was unsafe, whereas ≤ 25 mg L^–1^ NaF had harmless effects.

**Systematic Review Registration:**

PROSPERO registration No. CRD42022347357.

## INTRODUCTION

The human body has distinct habitats colonized by microbes, forming a functional and dynamic interface between our genes and the environment.^2^ Over recent decades, considerable attention has been given to the gut microbiota or microbiome that resides within the gastrointestinal tract.^3^ The Human Microbiome Project discovered that up to 70% (∼10^13^-10^14^ bacterial cells) of the body’s total microbiome (>39 trillion) reside in the gastrointestinal tract.^4–6^ These bacteria interact in commensal, symbiotic, or parasitic ways and are a source of competition for nutrition and adhesion toward some external pathogenic bacteria.^7^ Advances in molecular sequencing and computational methods have provided an unprecedented understanding of how the gut microbiota functions in symbiotically with the host, contributing to nutrition, metabolism, immune response, and intestinal architecture.^3^ Furthermore, targeting the gut microbiota with lifestyle interventions can result in significant changes in bacterial composition aligned with improvements in health conditions.^8^ Therefore, the relationship between the gut microbiota and human health appears to be bidirectional rather than consequential.^9^^,^^10^

There is not yet a single definition of a healthy microbiota. Nevertheless, it is mostly accepted that greater diversity, richness, and stability, and a higher relative abundance of species associated with the production of short-chain fatty acids (SCFAs) (eg, *Faecalibacterium prausnitziii*, *Bacteroides* spp, *Roseburia* spp, *Bifidobacterium* spp, and *Lactobacillus* spp) are hallmarks of a microbial community that is associated with better health outcomes.^11^ Specific bacteria shown to be more abundant in health compared with disease states include *Bifidobacterium* and *Lactobacillus* spp.^12^

Fluoride is well recognized for its role in preventing and reversing dental caries.^13^ Fluoride is naturally found in soil, water, and almost all food and drink items in different concentrations.^14^ Fluoride, often in the form of sodium fluoride (NaF), is added to dental products, as well as to water, salt, and milk, in many countries globally to prevent dental caries.^13^^,^^1^^,^^15^

Although fluoride has been widely used as a preventive agent for dental caries, the effects of fluoride on the gut microbial communities still need to be understood.^16^ Fluoride can affect microbial community abundance, which may enhance its ability to prevent the growth of harmful bacteria and fungi or stimulate the growth of useful bacteria that reside in the gastrointestinal tract and vice versa, as summarized by Moran et al^17^ in a mini-review in which they discuss recent studies in which the effects of ingested fluoridated water on human and animal microflora were observed, mainly focusing on oral microbiomes.^17^ When gut microbiome was discussed, it was mainly in animal models, with a focus on how acute toxicity of fluoride can perturb normal gut microbiomes. To our knowledge, no previously published systematic reviews have highlighted the impacts of fluoride with a specific target on gut microbiota and its associated functions. Fluoride may be beneficial or harmful to the gut microbiome depending upon the doses ingested, transit time, and duration of exposure.^1^^,^^18^ Levels of daily fluoride intake mainly depend on the geographic region and sources of exposure, with most people exposed to a range between 0.46 and 3.6-5.4 mg day^–1^.^19^ The daily adequate intakes of fluoride suggested by the US Environmental Protection Agency and World Health Organization (WHO) are 0.5-0.7 mg for infants, 1-2 mg for children, and 3-4 mg for adults males and females.^20^ The Environmental Protection Agency allows a range of 0.8-1.7 mg L^–1^ in the drinking water, with a tolerable upper intake level of 4 mg L^–1^.^21^

However, the optimal scale of fluoride in drinking water recommended by the WHO is 1.5 mg L^–1^, with the high or upper limit being 4 mg L^–1^ for humans to prevent dental caries and skeletal disorders.^17^^,^^22^ In 1984, WHO discovered that teeth mottling is associated with fluoride levels > 1.5 mg L^–1^ in water, and skeletal fluorosis can ensue if fluoride levels exceed 10 mg L^–1^. Therefore, 1.5 mg L^–1^ was recommended as a safe dose by WHO. However, this value is not fixed and should be adjusted by considering local conditions (eg, diet, water consumption).^23^^,^^24^ In diet, the dose of ≤ 5 mg kg^–1^ fluoride in the form of salt is regarded as the minimum but is dependent on age and sex.^23^^,^^24^ Doses effective for gut microbiota still need to be recognized; human intervention trials are relatively limited.

In this systematic review, we provide an overview of all recent studies that investigated the association between fluoride exposure from different sources, doses, and duration with changes in gut microbiome composition in animals, in vitro models, and humans. The primary outcome was to define any fluoride-induced changes in the composition of the gut microbiome, measured by (1) abundance and/or (2) diversity or richness of the microbes. The secondary outcome measured the significant impacts of dose and duration of fluoride on microbiota-associated functions (ie, metabolites and gene expression) in both animals and humans.

## METHODS

### Protocol and Registration

This systemic review was conducted according to the recommendations of the Preferred Reporting Items for Systematic Reviews and Meta-analysis (PRISMA) statement and the guidelines of the *Cochrane Handbook for Systematic Reviews of Interventions*.^25^^,^^26^ The full details of the processes including the eligibility criteria, search strategy, extraction process, and data analysis were prespecified and documented in a protocol that was registered in the PROSPERO database (registration no. CRD42022347357).

### Search Strategy

The search strategy was developed according to the research question, using the population, exposure, outcome, and study design (PEOS) approach listed in Table 1.

**Table 1.** Research question based on population, exposure, outcome, and study design terms.

Research question	What is the effect of fluoride exposure on gut microbial communities?
Population	All animals and humans. All animals have been considered. In vitro studies, such as fermentation models, have also been considered.
Exposure/intervention	Any source and form of fluoride: topical (eg, dental products) and systemic (eg, dietary and nondietary supplements, diet, and air)
Outcome	Abundance and/or diversity of the gut microbes, after fluoride exposure
Study design	All observational or analytical, quantitative, and laboratory-based or in vitro studies

A literature search was undertaken in July 2022 and a rerun of the search was conducted in July 2023 and then in 2024, using the following 6 electronic databases: MEDLINE, CINAHL, Embase, Scopus, PubMed, and Web of Science. Publications in English were retrieved using a combination of key terms, subject index, and Medical Subject Heading terms. A search of the reference lists of relevant articles was also performed to identify other potentially relevant sources. The terms used were “gut,” “gastrointestinal,” “intestine,” “colon,” “bowel,” “microbiota,” “micro biota,” “microbiome,” “micro biome,” “flora,” “microflora,” “micro flora,” “bacteria,” “fluorid,” “fluoride,” and “fluoridation.” All keywords and search strategies were adapted according to the specifics of each database and are presented in Tables S1 and S2.

There were no restrictions based on the date of publication. All published articles that met the stated eligibility criteria were included. Studies reported only in the English language were included, due to lack of translation facilities.

### Inclusion and Exclusion Criteria

The eligibility criteria were based on the PEOS framework (ie, population, exposure, outcome, study design). Based on the study design, all observational or analytical studies that attempted to identify the relationship between fluoride exposure to gut microbiota and an associated outcome, such as change in the composition of gut microbiota and its associated effects, were included. Quantitative studies using all interventional trials or designs, such as randomized controlled trials and nonrandomized controlled trials, along with laboratory-based studies (eg, fluoride exposure to in vitro models) were also considered. Before and after studies, also called pre-post studies, and multiarm studies were also considered for inclusion.

All studies of humans or other animals of any age and sex that were exposed to different forms and doses of fluoride, in which gut microbiome speciation analysis was conducted using DNA analysis and gene sequencing to determine the effect of systemic fluoride on participants’ gut microbiome, were selected. Additionally, studies using in vitro models created from fecal or gut samples to evaluate the effect of fluoride were also included.

Exposure or intervention included fluoride in all forms and sources: topical (eg, dental products) and systemic (eg, diet, dietary and nondietary supplements, air). The outcome was the evaluation of microbiota composition after fluoride exposure, including the assessment of diversity or richness, prevalence of bacterial taxa, and their corresponding functions, such as metabolic and gene expressions.

Diversity or richness was evaluated by considering alpha and beta diversity values. Three diversity scales or indices were estimated for alpha diversity: Shannon diversity index, Simpson diversity index, and Chao 1 index. The Shannon diversity index is used to measure both the operational taxonomic units of abundance or richness, and evenness of species.^27^ The Simpson diversity index is another measure of diversity; it measures the relative abundance of each species and gives more weight to the more abundant species in the sample.^28^ Chao 1 is an estimator for total species richness that gives more importance to unique species.^29^

Qualitative studies such as case reviews, case series, expert opinions, and reviews, focus groups and interviews, narrative reports, abstracts without full text, and conference papers, as well as other systematic reviews and study protocols, were excluded.

### Study Selection

After the search stage, all the citations were exported to Endnote 21.2 (Clarivate, London, UK) and deduplicated. The remaining studies were then exported to Covidence 2022 software (Melbourne, VIC, Australia) for screening. The Covidence software was also useful for removing duplicates that were not identified in Endnote.

The abstract and title of each paper were screened independently for eligibility by 2 individuals (M.Y. and M.P.S.) according to the eligibility requirements. In case of disagreement, the conflicts were resolved by discussion with another reviewer (F.V.Z., C.H.O., and P.D.). Full text of seemingly relevant articles was then screened by 2 reviewers independently (M.Y. and M.P.S.) to determine their eligibility for inclusion in the final review. Again, any conflicts were resolved by discussion with a third reviewer. All missing full texts were requested via ResearchGate and/or the university library to allow access to the full text. Corresponding authors were contacted to request full-text articles not otherwise available. Full-text articles that could not be accessed after following these steps were excluded.

Manual searching was also done to exhaust all possibilities and to reduce the risk of bias, as follows: (1) reference lists from the included studies were searched to identify any relevant study; (2) performing citation tracking in which all the articles that cited each of the included articles were tracked; and (3) related articles in Google Scholar and Science Direct were searched for to prevent any chance of missing a relevant study. The relevant articles underwent further scrutiny against the inclusion criteria, after title and abstract and full-text screening.

### Data Extraction

A bespoke tool was developed in Covidence that included the study identifier, author, year of publication, journal, title, study location, study design, study period, details of exposure, study participants, number of participants analyzed, outcomes (methods for measuring outcome), statistical analysis method, and software used. Data extraction was conducted by 1 reviewer (M.Y.) and cross-checked by another reviewer (E.A.K.). Differences in judgment between the 2 reviewers were settled by discussion and consensus.

### Quality Assessment

The Mixed Methods Appraisal Tool was used to assess the quality of the included studies.^30^ The Mixed Methods Appraisal Tool contains 5 and 6 evaluation elements/questions each for the various study designs, with a judgement of “yes,” “no,” and “unclear” for each question.

### Data Synthesis and Analysis

All the data are presented in a narrative form using figures and tables to assist in data presentation by following PRISMA guidelines. The supplementation and control groups’ pre- and postintervention mean values and SDs were aggregated in the form of tables. Subgroup analysis was conducted based on dose and duration.

## RESULTS

### Identification and Selection of Studies

**Figure 1.**
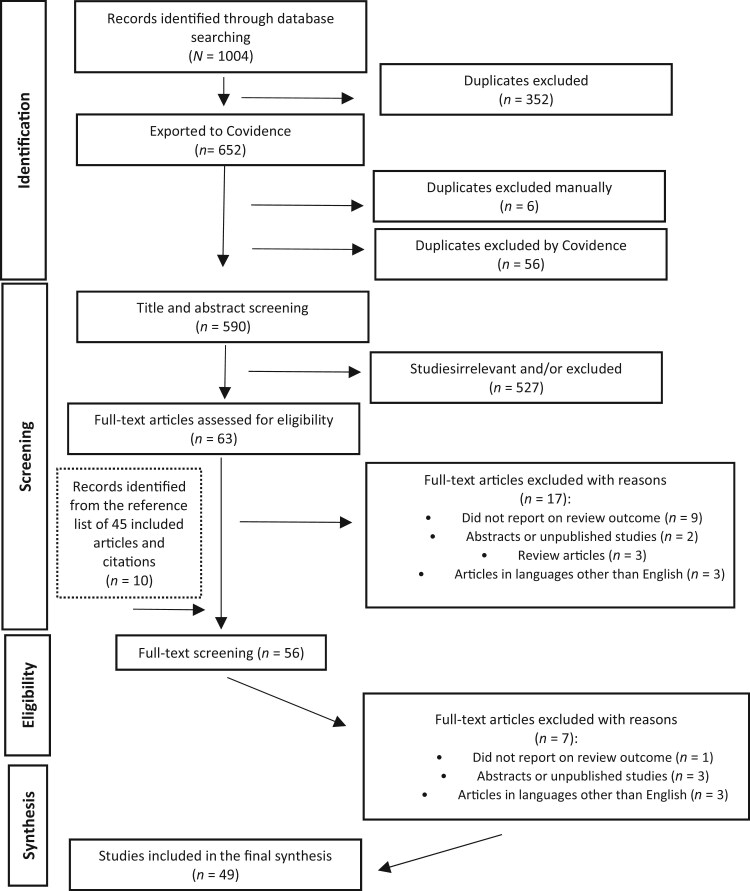
PRISMA flow diagram for screening of studies

Study identification and selection are specified in the PRISMA flow chart (Figure 1). A total of 1004 articles were identified from the database search. After deduplication 590 records were screened at the title and abstract level, and 63 articles were included in the full-text screening based on the preset inclusion and exclusion criteria. An additional 10 articles were identified from reference lists and citation chaining of included studies. Of the 73 remaining articles, 49 were eligible for and included in this review. All 49 articles reported on studies of the effect of different forms and doses of fluoride on the gut microbiome. These studies were 39 randomized controlled trials,^10^^,^^31–67^ 1 nonrandomized controlled trial,^68^ 6 laboratory-based or experimental studies,^14^^,^^69–73^ 1 cohort study,^74^ and 2 case-control studies.^75^^,^^76^ The principal aim differed among the studies. The details of studies are given in Tables 2 and 3,^10^^,^^14^^,^^31–76^ and Table S3.

**Table 2.** Summary of characteristics of included studies.

Classification	No. of studies (frequency [%])
Year of publication	
2000 or earlier	0 (0)
2001-2010	0 (0)
2011-2020	21 (44)
After 2020	28 (56)
Distribution, by region, of the included studies	
Asia	44 (90)
Europe	0 (0)
North America	2 (4)
South America	1 (2)
Australia	2 (4)
Study design	
Cohort	1 (2 )
Laboratory based	6 (13)
RCT	39 (79)
Non-RCT	1 (2.1)
Cross-sectional	0 (0)
Case-control study	2 (4)
Animal study	42 (88**)**
Type of animal	
Kunming mice	3 (6)
ICR mice	5 (10)
*Bombyx mori* (silkworm)	3 (6)
Sprague-Dawley rats	6 (13)
Wistar rats	3 (6)
Bovine	2 (4)
*Drosophila melanogaster* (fruit fly)	1 (2.1)
*Bufo gargarizans* tadpoles	1 (2.1)
Ducklings	1 (2.1)
Broiler chicken	1 (2.1)
Laying hens	2 (4)
Wild-type BALB/c mice	1 (2.1)
Common carp	1 (2.1)
Zebrafish	1 (2.1)
Offspring rats	4 (8)
Mice	3 (6)
Inbred male C57BL/6 J mice	4 (8)
Age group	
24 h-1wk	4 (10)
2 wk-3 mo	17 (40)
4 mo to ≥8 mo	2 (5)
Not reported	19 (45)
Sex	
Male	19 (45)
Female	8 (19)
Both male and female	3 (7)
Not reported	12 (29)
Biomarker	
Fecal	16 (38)
Intestinal tissue	6 (14)
Duodenum and colon content	1 (2)
Cattle rumen	2 (5)
Ileum	4 (8)
Gut	2 (5)
Small intestine and cecal content	3 (5)
Rectal content	1 (2)
Kidney and colon	1 (2)
Colon	6 (14)
Human study	4 (8)
In vitro model	3 (6)
Age group, y	
0-20	1 (25)
21-40	1 (25)
>40	0 (0)
Not reported	2 (50)
Sex	
Male	0 (0)
Female	1 (25)
Both male and female	1 (25)
Not reported	2 (50)
Biomarker	
Fecal	4 (100)
Intestinal tissue	0 (0)
Colon content	0 (0)

Abbreviations: BALB/c, Bagg albino; ICR, Institute of Cancer Research; nRCT, nonrandomized controlled trial; RCT, randomized controlled trial.

### Characteristics of Included Studies

The majority (90%) of the studies were conducted in Asia (China, Turkey, India, and Japan),^10^^,^^14^^,^^31–33^^,^^35–43^^,^^46–52^^,^^54–68^^,^^70–76^ 4% in the United States,^45^^,^^53^ 4% in Australia,^34^^,^^69^ and 2% in Brazil (Table 2).^44^ Forty-two^10^^,^^31^^,^^33–70^^,^^76^ studies (88%) were conducted with animals, 3 were in vitro (6%),^71–73^ and 4 studies (8%)^14^^,^^74–76^ included humans as the participants (Table 2). Zhou et al^76^ investigated the changes in the gut microbiome of children with dental fluorosis and compared them with a mouse model established by administering 100 mg L^–1^ NaF in water. This study, therefore, was considered both an animal and human study.

The included studies used different animal models, such as rodents, birds, and fish (Table 2). The sample size in the case of animal studies ranged from 6 to 900 animals. In the case of human studies, it ranged from 15 to 114 participants. All human^14^^,^^74–76^ and 16 (40%)^33^^,^^36^^,^^40^^,^^45–47^^,^^52–54^^,^^56^^,^^57^^,^^60^^,^^65–67^^,^^76^ animal studies included an analysis of microbiota on fecal samples. The remaining 26 animal studies used other tissues as biomarkers (Table 2).

Various sequencing workflows were used in the reviewed literature to estimate gut microbiota composition. Amplicon 16S rRNA gene sequencing was used in 44 studies (92%), 2 studies^41^^,^^46^ also included quantitative polymerase chain reaction to validate 16S rRNA gene sequencing findings. Six studies^50^^,^^51^^,^^55^^,^^58^^,^^67^^,^^71^ used real-time–quantitative polymerase chain reaction using 16S rRNA-specific primers to quantify bacterial species. Table 3 presents full details of reviewed and included articles.

**Table 3.** Summary of outcomes in the included study^a^

Reference (first author, year); country	Fluoride dose	Gut microbiome estimation (method)	Microorganism		Metabolite		Methods used for assessing microbial richness and results				Microbial alpha diversity method and results			
			**Control group** ^b^	**Intervention group** ^c^	**Control** ^b^	**Intervention** ^c^	OTU		Chao 1 index		Simpson diversity		Shannon diversity	
							Control	Treated	Control	Treated	Control	Treated	Control	Treated
Davis, 2012^69^; Australia	20 mM fluoroacetate (added in media)	16S rRNA gene sequences were aligned with the Green Genes alignment tool ANOVA	N/A	Synergistetes ↑ *Enterococcus* spp ↑	Glycerol, lactate, ethylene glycol, citrate, ethanol	Glycerol, lactate, ethylene glycol, citrate, ethanol	N/A	N/A	N/A	N/A	N/A	N/A	N/A	N/A
Ma, 2014^71^; China	NaF: 0.1, 1, 10, and 100 mM Supplemented in media	Trizol reagent RNA extraction qRT-PCR to validate differentially abundant genera	*Escherichia coli*	More fluorescent at low concentrations than higher	N/A	N/A	N/A	N/A	N/A	N/A	N/A	N/A	N/A	N/A
Li, 2016^39^; China	200 mg kg^–1^ NaF solution	Bacterial DNA kit Bacteria: 16S rRNA gene sequencing- Illumina MiSeq platform FLASH scRNA-seq method was used for demultiplexing and quality filtering QIIME (v. 1.7) was used to cluster OTUs applying a cutoff of 97% similarity	Firmicutes, Proteobacteria, Bacteroidetes, Cyanobacteria Fusobacteria, Chloroflexi, Euryarchaeota, Thaumarchaeota	Firmicutes ↓ Proteobacteria ↓ Thaumarchaeota Euryarchaeota ↑	Acetic acid Propionic acid Butyric acid Isobutyric acid Isovaleric acid	Isobutyric acid Isovaleric acid Acetic acid ↑ Propionic acid ↑ Butyric acid ↑	**Fluoride resitant strain T6:** 5040 **Fluoride susceptible strain 734: **5737	**Fluoride resitant strain T6:** 5207 Fluoride susceptible strain 734: 6136	**Fluoride resitant strain T6:** 11 403.44 **Fluoride susceptible strain 734:** 12373.26	**Fluoride resitant strain T6:** 11 665.57 **Fluoride susceptible strain 734: **14 024.97	N/A	N/A	**Fluoride resitant strain T6**: 5.53 **Fluoride susceptible strain 734: **5.99	**Fluoride resitant strain T6: **5.62 **Fluoride susceptible strain 734:** 6.33
Luo, 2016^41^; China	0, 400, 800, 120 mg kg^–1^ F	Phenol chloroform extraction of DNA followed by bead beating qPCR to validate differentially abundant genera	*Lactobacillus* spp *Bifidobacterium* spp *Escherichia coli* *Enterococcus* spp *L salivarius, Clostridium spiroforme, Streptococcus lutetiensis* *Weissella hellenica*	**400 mg F kg^–1^**: *E coli* ↑ *Enterococcus* spp ↑ *Lactobacillus* spp ↓ *Bifidobacterium* spp ↓ **800 mg F kg^–1^**: *Lactobacillus* spp ↓ *Bifidobacterium* spp ↓ *E coli* ↑ *Enterococcus* spp ↑ **1200 mg F kg^–1^**: *Lactobacillus* spp ↓ *Bifidobacterium* spp ↓ *E coli* ↑ *Enterococcus* spp ↑	N/A	N/A	N/A	N/A	N/A	N/A	N/A	N/A	N/A	N/A
Yasuda, 2017^53^; United States	4 ppm F in drinking water 4 ppm F + 2.25 μg d^–1^ F via gavage	DNA extraction by MP Bio Fast DNA Spin kit for soil 16srRNA sequencing by Illumina MiSeq platform QIIME version was used to quality filter unique reads and clustered into OTUs at a 97% similarity level using Greengenes, version 2013.	*Streptococcus* spp Pasteurellaceae *Bacteroides*, Clostridiales Lachnospiraceae *Parabacteroides distasonis* *Bacteroides uniformis* *Bacteroides*	**0 wk (oral)** *Streptococcus* spp Pasteurellaceae **0 wk (gut)** Bacteroides Clostridiales Lachnospiraceae *Parabacteroides* **12 wk (oral)** *Parabacteroides distasonis* *B uniformis* *Sutterella* *Bilophila* Bacteroidales *Burkholderia* **12 wk (gut):** No change observed	N/A	**4 ppm F:** Glyoxylate cycle ↓ Succinate dehydrogenase ↓ mevalonate ↓ **4 ppm F + 2.25 μg d^–1^ F via gavage:** Glyoxylate cycle ↓ Succinate dehydrogenase↓ Mevalonate ↓	N/A	10	N/A	N/A	N/A	N/A	N/A	N/A
Dutta, 2020^35^; India	100 µg mL^–1^ NaF (52 µg mL^–1^ F) 200 µg mL^–1^ NaF (89.9 µg mL^–1^ F) 300 µg mL^–1^ NaF (126 µg mL^–1^ F) 400 µg mL^–1^ NaF (157 µg mL^–1^ F) 500 µg mL^–1^ NaF (175 µg mL^–1^ F)	DNA extraction using Marmur’s protocol Sequence alignment by CLUSTRAL Phylogenetic tree was constructed using TREECON software	KT201599 *Bacillus* KT201600 *Bacillus*	KT201599 *Bacillus* ↑ KT201600 *Bacillus* ↑ Enhanced bacterial growth as the dose increased	N/A	N/A	N/A	N/A	N/A	N/A	N/A	N/A	N/A	N/A
Parthasaradhi, 2018^72^; India	NaF concentration (mM): 10.0, 20.0, 30.0, 40.0, 50.0	Culturing using microdilution	*L salivarius* and *L acidophilus*	*L salivarius* and *L acidophilus* inhibited as the dose increased	N/A	N/A	N/A	N/A	N/A	N/A	N/A	N/A	N/A	N/A
Cao, 2020^33^; China	100mg L^–1^ NaF in water	Power Soil DNA kit following “standard” protocol Illumina Hiseq 2500 platform Trimmomatic(v. 0.33) was used for quality filtering. QIIME was used for OTUs.	Ascomycota 63.84% Basidiomycota 9.94% Mortierellomycota 1.51% Chytridiomycota 0.21% Glomeromycota 0.06% Eurotiomycetes 28.86% Dothideomycetes 20.66% Sordariomycetes 15.10% *Penicillium* 18.29% *Alternaria* 14.76% *Aspergillus* 8.37%	Ascomycota 76.02% Basidiomycota 7.76% Mortierellomycota 2.26% Chytridiomycota 0.34% Glomeromycota 0.23% Eurotiomycetes 24.58% Dothideomycetes 29.50% Sordariomycetes 15.10% *Penicillium* 7.45% *Alternaria* 23.36% *Aspergillus* 12.70% Ustilaginomycetes *Microdochium* *Plectosphaerella* *Pluteus*	N/A	N/A	305	154	N/A	N/A	N/A	N/A	N/A	N/A
Fu, 2020^36^; China	100 mg L^–1^ NaF	Power Soil DNA kit following “standard” protocol 16SrRNA sequencing Illumina Hiseq 2500 platform QIIME (v. 1.8.0) pipeline was used for quality filtering, and sequences with 97% similarity of OTU were clustered using SILVA. *t* Test for relative specie abundance	Firmicutes 56.04% Bacteroidetes 40.56% Verrucomicrobia 1.82% Proteobacteria Tenericutes Actinobacteria Saccharibacteria Cyanobacteria Bacteroidales_S24-7 36.67% *Lactobacillus* 7.35% *Faecalibaculum* 33.66%	Firmicutes 41.98% Bacteroidetes 54.52% Verrucomicrobia 1.20% Proteobacteria Tenericutes ↑ Actinobacteria Saccharibacteria Cyanobacteria Bacteroidales *Lactobacillus* *Alloprevotella* (*Eubacterium* ↑) *Alloprevotella* ↑ Prevotellaceae ↑ *Ruminiclostridium* 9 ↑ *Faecalibaculum* ↓	N/A	GSH activity ↓ SOD activity↓ CAT ↓	3	22	N/A	N/A	N/A	N/A	N/A	N/A
Liu, 2019^31^; China	110.5 mg NaF (50 mg L^–1^ F ion) 221 mg NaF (100 mg L^–1^ F ion)	DNA sequencing- Illumina MiSeqSystem Trimmomatric and FLASH pipeline was used for quality filtering After trimming, unique sequences were further denoised using a preclustering algorithm, chimeras were removed using UCHIME.	Firmicutes 88.99% Bacteroidetes 5.61% Saccharibacteria 2.86% Proteobacteria 1.19% Actinobacteria 1.14% *Lactobacillus* 72.43% Lachnospiraceae 5.62% Bacteroidales 3.78% Lachnospiraceae 2.28% *Candidatus Saccharimonas* 2.86%	**221 mg NaF (100 mg L^–1^ F ion)** Firmicutes 77.27%; *P* = .0317 Bacteroidetes 13.69%; *P* = .04462 Saccharibacteria 2.37% Proteobacteria 3.28% Actinobacteria 2.65% *P* = 0.01085 *Lactobacillus* 48.02% Lachnospiraceae 8.49% Bacteroidales 8.53% norank_f_Lachnospiraceae 4.03% *Candidatus Sacchar-imonas* 2.37%	Glycoproteins	Glycoproteins ↓	566	620	616.1	684	0.165	0.065	3.108	4.045
Pimentel, 2019^44^; Brazil	0.266 mg kg^–1^ sodium fluoroacetate	Phenol chloroform extraction of DNA	*Enterococcus*	*Enterococcus* resistant to sodium fluoroacetate	N/A	N/A	N/A	N/A	N/A	N/A	N/A	N/A	N/A	N/A
Wang, 2019^49^; China	NaF (mg L^–1^): 0.5, 5, 50	PowerSoil DNA Isolation Kit (MoBio), following “standard” protocols 16S rRNA PCR IlluminaMiSeq USEARCH and QIIME version 1.8 was used to quality filter unique reads. Reads were clustered into OTUs at a 97% similarity level using UCLUST algorithm.	Fusobacteria 41.67% Bacteroides Proteobacteria 22.28% Firmicutes	**0.5 mg L^–1^ NaF:** Fusobacteria 29.48% Bacteroidetes Proteobacteria 26.96% Firmicutes *Raoultella* *Shewanella* *Escherichia-Shigella* Lachnospiraceae Uncultured_f_Porphyromonadaceae *Ruminococcus* *Lachnoclostridium* 5 **5 mg L^–1^ NaF:** Fusobacteria 37.36%, Bacteroidetes Proteobacteria 24.76% Firmicutes *Ruminococcus* *Lachnoclostridium* 5 **50 mg L^–1^ NaF:** Fusobacteria 20.97% Bacteroidetes/Firmicutes Proteobacteria 40.12%	N/A	Energy metabolic pathways downregulated	79	**0.5 mg L^–1^ NaF:** 93 **5 mg** L**^–1^ NaF:** 106 **50 mg L^–1^ NaF:** 166	N/A	N/A	N/A	N/A	N/A	N/A
Li, 2020^70^; China	4.76 mM NaF	TIANampBacteria DNA Kit following manufacturer instructions Bacteria: 16S rRNA gene Illumina sequencing RNA extraction by Trizol reagent	*Enterrococcus faecalis* TV 4	*E faecalis* TV 4 ↓	N/A	Casease Lipase Amylase	N/A	N/A	N/A	N/A	N/A	N/A	N/A	N/A
Miao, 2020^42^; China	400 mg kg^–1^ F (low F) 1200 mg kg^–1^ F (high F)	QIAamp DNA Stool Mini Kit (QIAGEN), following manufacturer instructions. RNA extraction by Trizol reagent 16S rRNA illumine sequencing QIIME (version not specified) was used to quality filter, denoise, and analyze sequences, which were assigned to OTUs using SILVA, with a threshold of 97% pairwise identity and classified taxonomically	Bacteroidetes Firmicutes Proteobacteria Acidobacteria Chloroflexi, Actinobacteria, *Lactobacillus*	**400 (low F) mg kg^–1^ F:** Bacteroidetes Firmicutes Proteobacteria ↓ **1200 mg kg^–1^ F (high F):** Bacteroidetes, Firmicutes, Proteobacteria ↑ Acidobacteria ↑ Chloroflexi ↑ Actinobacteria ↑ Chloroflexi Gammaproteobacteria, *Escherichia*, *Shigella* Streptococcaceae Enterobacter	d-lactate DAO IL-1β IL-6 TNF-α ZO-1 Claudin-1 Claudin-4 Acetic acid Propionic acid Butyric acid Iso-pentanoic acid Isobutyric acid Pentanoic acid	**400 (low F) mg kg^–1^ F:** No change observed **1200 mg kg^–1^ F (high F):** d-lactate ↑ DAO ↑ IL-1β ↑ IL-6 ↑ TNF-α ↑ ZO-2 ↓ Claudi-4 ↓ ZO-1 ↓ Claudin-1 ↓ Claudin-4 ↓ Acetic acid ↓ Propionic acid ↑ Butyric acid ↓ Iso-pentanoic acid ↓ Isobutyric acid ↓ Pentanoic acid	2959	N/A	N/A	N/A	Increased in high F group	Increased in high F group	N/A	N/A
Miao, 2020^43^; China	400 mg kg^–1^ F (low F) 1200 mg kg^–1^ F (high F)	RNA extraction by Trizol reagent QIAamp DNA Stool Mini Kit (QIAGEN), following manufacturer instructions Bacteria: 16S rRNA gene sequencing Illumina Phylogenetic affiliation using SILVA reference database Chimeras were removed using USEARCH.	*Lactobacillus*	**1200 mg kg^–1^ F (high F):** Gammaproteobacteria ↑ Streptococcaceae ↑ Enterobacteriales ↑ Enterobacteriaceae ↑ *Escherichia-Shigella* ↑	Amylase Maltase Lactase Lipase Trypsin Sucrase	**1200 mg kg^–1^ F (high F):** Amylase ↓ Maltase ↓ Lactase ↓ Lipase Trypsin Sucrase	105	**400 mg kg^–1^ F (low F): **88 **1200 mg kg^–1^ F (high F): **853	N/A	N/A	N/A	N/A	N/A	N/A
Parthasaradhi, 2020^73^; India	NaF concentration (mM): 10.0, 20.0, 30.0, 40.0, 50.0	Bradford protein assay and SDS-PAGE	*L salivarius* and *L acidophilus*	N/A	Enolase enzyme	Enolase enzyme ↓	N/A	N/A	N/A	N/A	N/A	N/A	N/A	N/A
Qiu, 2020^45^; United States	100 mg L^–1^ NaF	Fecal DNA was extracted with QIAamp DNA Stool Mini Kit (QIAGEN), following manufacturer instructions. 16S rRNA gene sequencing Illumina MiSeq platform	Firmicutes (70.3%-81.1%) Bacteroidetes (13.4%-23.3%) Proteobacteria (0.8%-2.7%) Patescibacteria (0.2%-1.3%) Actinobacteria (1.0%-1.2%) Tenericutes (0.6%-1.0%) Cyanobacteria Verrucomicrobia, Epsilonbacteraeota, *Gastranaerophilales*	Peptococcaceae ↑ Rikenellaceae ↑ Peptococcaceae	N/A	N/A	456	542	193.32 ± 7.34	152.80 ± 20.81	N/A	N/A	6.02 ± 0.31	6.25 ± 0.14
Sun, 2020^46^; China	100 mg L^–1^ NaF 100 mg L^–1^ NaF and *L johnsonii* BS15 probiotic	Total RNA kit for RNA extraction DNA isolated by stool DNA isolation kit qPCR	*L johnsonii* BS15, Enterobacteriaceae *Lactobacillus* spp Bacteroidetes Firmicutes	**100 mg L^–1^ NaF:** *L johnsonii*BS15 Enterobacteriaceae *Lactobacillus* spp ↓ Bacteroidetes Firmicutes **100 mg L^–1^ NaF and *L johnsonii* BS15 probiotic:** *L johnsonii BS15* ↑ Enterobacteriaceae ↑ *Lactobacillus* spp Bacteroidetes Firmicutes **After 105 d or 70 d:** Enterobacteriaceae ↓ *Lactobacillus* spp ↑	T-AOC GSH-Px SOD MDA Amylase Trypsin Lipase	**100 mg L^–1^ NaF:** T-AOC ↓ GSH-Px ↓ SOD MDA ↑ Amylase ↓ Trypsin ↓ Lipase ↓ Secretory IgA ↑ **100 mg L^–1^ NaF and *L johnsonii* BS15 probiotic:** T-AOC GSH-Px SOD↑ MDA ↓	N/A	N/A	N/A	N/A	N/A	N/A	N/A	N/A
Wang, 2020^48^; China	50 and 100 mg L^–1^ F	Kit for DNA extraction 16S rRNA gene sequencing Illumina MiSeq FLASH was used for merging reads and sequences. UPARSE was used to cluster reads at 97% similarity.	*Lactobacillus* 43.97% Bacteroidales 14.06% Actinobacteria 1.82% Unclassified Coriobacteriaceae 0.33% *Oscillibacter* 0.07% Unclassified Prevotellaceae 0.09%	**100 mg** L^–1^ **F:** Firmicutes ↓ 15.65%Saccharibacteria ↓ 5.16% Actinobacteria ↓ 0.67% Bacteroidetes ↑ 15.33 Proteroidetes ↑ 7.05% *Lactobacillus* 23.49% norank_f_Bacteroidales_S24-7 ↑ 17.93% Verrucomicrobia 0.003% Unclassified_f_Coriobacteriaceae 0.06% *Oscillibacter* 0.32% unclassified_f_Prevotellaceae 0.27%	Glycoproteins	**100 mg L^–1^ NaF:** Glycoproteins ↓ 29.13% **50 mg L^–1^ F:** Glycoproteins ↓ 13.20%	N/A	N/A	N/A	N/A	N/A	N/A	N/A	N/A
Shi, 2020^66^; China	0.5 mg kg^–1^ (mg L^–1^) PFOA 1 mg kg^–1^ (mg L^–1^) PFOA 3 mg kg^–1^ (mg L^–1^) PFOA	16S rRNA sequencing	Bacteroidetes Firmicutes Actinobacteria Proteobacteria Cyanobacteria *Bifidobacterium pseudolongum* *Anoxybacillus kestanbolensis* *Gemmiger formicilis* *Bifidobacterium bifidum* *Ruminococcus gnavus* *A muciniphila*	Bacteroidetes ↑ Firmicutes ↓ Actinobacteria ↓ Proteobacteria ↓ *B pseudolongum* ↓ *A kestanbolensis* ↓ *G formicilis* ↓ *B bifidum* ↓ *R gnavus* ↓ *A muciniphila* ↓	FFAR2 GPR109 ZO-1 Occludin IL-1β, IL-6, TNF-α Tlr4	FFAR2 ↓ GPR109 ↓ ZO-1 ↓ Occludin ↓ IL-1β, IL-6, TNF-α ↑ Tlr4 ↑	N/A	N/A	N/A	N/A	N/A	N/A	N/A	N/A
Dionizio, 2021^34^; Australia	10 and 50 mg L^–1^ F	ZR Fungal/Bacterial DNAMicroPrep Kit Taxonomy was assigned to OTUs (97% clustering) using the SILVA (v. 138) database. Phangorn package (v. 2.5.5) was used for phylogenetic reconstruction	Campylobacteria Clostridia Gamma proteobacteria Bacilli Firmicutes Desulfobacterota	**10 mg L^–1^ F** Clostridia ↓ **50 mg L^–1^ F** *Ureaplasma* ↓	N/A	**10 mg L^–1^ F:** 276 Proteins identified **50 mg L^–1^ F:** 285 Proteins identified, including reduction in dystrophin and calcium/calmodulin-dependent protein kinase 1	N/A	453	N/A	N/A	N/A	N/A	N/A	N/A
Komuroglu, 2021^32^; Turkey	100 mg L^–1^ NaF	Gene MATRIX Tissue & Bacterial DNA Purification kit for total DNA extraction QIIME2 procedure for analyses of the 16S rRNA gene	Proteobacteria Firmicutes Bacteroidetes Actinobacteria Lactobacillales 55.5% *Lactobacillus* 50.9%	Proteobacteria ↑ Firmicutes ↓ Bacteroidetes ↓ Actinobacteria ↓ Lactobacillales ↓ 14.47% Pseudomonadaceae Myocoplasmataceae *Lactobacillus* 4.23%	MDA SOD Catalase	MDA ↑ SOD ↓ Catalase ↓								
Li, 2021^38^; China	750mg kg^–1^ NaF in feed	QIAamp DNA Mini Kit for DNA extraction QIIME (v. 1.9.0) was used to collapse de novo OTUs at 97% identity.	Proteobacteria Firmicutes Bacteroidetes Actinobacteria Lactobacillales 55.5% *Lactobacillus* 50.9%	Firmicutes 76.19%, Bacteroidetes 20.48 Proteobacteria 0.03% Bacteroides 20.24% Uncultured bacterium Lachnospiraceae 19.83% *Turicibacter* 14.78%	N/A	N/A	300	97	401.13 ± 34.04	273.40 ± 81.53	5.47 ± 0.52	4.51 ± 1.71	0.91 ± 0.06	0.83 ± 0.19
Liu, 2021^40^; China	100 mg L^–1^ NaF	Trizol reagent RNA extraction Chimeras were removed using UCHIME.	Actinobacteria Firmicutes Bacteroidetes Proteobacteria	Parabacteroides ↑ *Oscillospira* ↑ *Dehalobacterium* ↑ *Lactobacillus* ↓ SMB53 ↓ *Phascolarctobacterium* ↑	FSH LH Testosterone	FSH LC3-II/LC3-I ↑ p62 ↓ Beclin1 ↑	N/A	N/A	N/A	N/A	N/A	N/A	N/A	N/A
Xin, 2021^50^; China	100 mg L^–1^ NaF 100 mg L^–1^ NaF and *L johnsonii* BS15 probiotic	RNA extraction using Total RNA kit RT-qPCR Shapiro-Wilk normality test for RT-qPCR analysis DNA extraction by stool DNA isolation with manufacturer instructions Cutadapt was used to quality-filter unique reads. Reads were clustered into OTUs at a 97% similarity level using Uparse v. 7.0.1001 and phylogenetic analysis by muscle software.	Firmicutes 80.3% Bacteroidetes 12.9% Actinobacteria Proteobacteria, Tenericutes Melaninabacteria *Lactobacillus* 54.6% *Bacteroides* 2.1% *Dubosiella* 8.2% *Helicobacter* 4.1% Unidentified Lachnospiraceae 1.6% *Carnobacterium* *L intestinalis* *Carnobacterium maltaromaticum* *L reuteri* Firmicutes bacterium *L animalis*	**100 mg L^–1^ NaF:** Firmicutes 37.1% Bacteroidetes 52.8% Bacteroides 19.9% *Lactobacillus* 2.9% *Dubosiella* 2.5% *Helicobacter* 5.4% Unidentified Lachnospiraceae 4.0% **100 mg L^–1^ NaF and *L johnsonii* BS15 probiotic:** Firmicutes 68.7% Bacteroidetes 25.9% *Lactobacillus* 46.2% Bacteroides 11.5% *Dubosiella* 2.9% *Helicobacter* 2.0% Unidentified Lachnospiraceae 3.0%	*N*-acetyl-β-d-glucosaminidase	100 mg L^–1^ NaF *N*-acetyl-beta-d-glucosaminidase	20	**100 mg L^–1^ NaF: **86 **100 mg L^–1^ NaF and L johnsonii BS15 probiotic: **18	N/A	N/A	N/A	N/A	N/A	Increased
Xin, 2021^51^; China	100 ppm NaF ≈ 37.8 ± 2.4 ppm F ^–^ *L johnsonii* BS15 (probiotic group; 0.2 mL day^–1^	RNA extraction by E.Z.N.A. Total RNA Kit (OMEGA Bio-Tek) RT-qPCR and 16S rRNA sequencing Cutadapt was to quality-filter unique reads. 6S rRNA gene read pairs were demultiplexed based on the unique molecular barcodes, and reads were merged using VSEARCH.	Firmicutes Actinobacteria Bacteroidetes Cyanobacteria *Lactobacillus* Bacilli Lactobacillales Lactobacillaceae, *L taiwanensis* *L reuteri* *L intestinalis*	**100 ppm NaF ≈ 37.8 ± 2.4 ppm F ^–^** Firmicutes ↓ Actinobacteria ↑ Bacteroidetes ↑ Cyanobacteria ↑ Lactobacillus ↓ *Candidatus arthromitus* ↓ *Streptococcus* ↑ *Romboutsia* ↑ *Allobaculum* ↑ Unidentified Clostridiales ↑ *Dubosiella* ↑ *Bifidobacterium* ↑ Unidentified Lachnospiraceae ↑ ***L johnsonii* BS15 (probiotic group; 0.2 mL d^–1^)** *Streptococcus* *Romboutsia* *Allobaculum* Unidentified Clostridiales *Dubosiella* *Bifidobacterium*	BDNF CREB, cAMP response element-binding protein NCAM SCF mRNA PLP MOG MBP MAG Bcl-2 Bcl-xl Bax Bad Caspase 3 (highest) Caspase9 ZO-1 Claudin-1 Occludin d-lactate (lower)	**100 ppm NaF ≈ 37.8 ± 2.4 ppm F ^–^** BDNF ↓ CREB, cAMP response element-binding protein NCAM SCF mRNA ↓ PLP ↓ MOG MBP MAG ↓ Bcl-2 Bcl-xl ↓ Bax ↑ Bad ↑ Caspase3 Caspase 9 (highest) ZO-1 ↓ Claudin-1↓ Occludin ↓ d-lactate ↑ ***L johnsonii* BS15 (probiotic group; 0.2 mL d^–1^)** Claudin-1↓ Occludin ↓	N/A	N/A	N/A	N/A	N/A	N/A	N/A	N/A
Yan, 2021^52^; China	100 mg L^–1^ NaF (F group)	DNA extraction by QIAamp DNA Stool Mini Kit (Qiagen, Hilden, Germany) according to the manufacturer's protocols 16S rRNA gene sequencing Illumina MiSeq platform QIIME version 2019.1 was used to quality-filter unique reads and cutadapt (v. 2.8)to demultiplex sequences. Reads were clustered into OTUs at a 97% similarity level usingQIIME2	Firmicutes (62.4%-88.5%) Bacteroidetes (6.1%-33.3%) Proteobacteria 1.72% Actinobacteria 1.1% Tenericutes 0.8% Patescibacteria 0.7% Cyanobacteria Verrucomicrobia Epsilonbacteraeota Ruminococcaceae 3.1% Lachnospiraceae 19.5% Lactobacillaceae 17.6% Muribaculaceae 17.5% Erysipelotrichaceae 6.0%	Proteobacteria Actinobacteria Tenericutes Patescibacteria Cyanobacteria Verrucomicrobia Epsilonbacteraeota Ruminococcaceae Lachnospiraceae Lactobacillaceae Muribaculaceae Erysipelotrichaceae	N/A	N/A	456	542	N/A	N/A	N/A	N/A	N/A	Higher
Yu, 2021^55^; China	80 mg L^–1^ NaF	RNA isolation by Trizol reagent qRT-PCR DNA isolation by QIAamp DNA Stool Minikit 16s rRNA gene sequence by IIIumina HiSeq2500 platform	Proteobacteria Fusobacteria Firmicutes Planctomycetes Bacteroidetes Actinobacteria *Citrobacter* *Akkermansia* *Roseomonas* *Aurantimicrobium*	Proteobacteria ↓ Fusobacteria ↑ Firmicutes ↑ Verrucomicrobia ↑ Bacteroidetes Actinobacteria ↓ Planctomycetes ↓ *Plesiomonas* ↑ *Citrobacter* ↓ *Akkermansia* ↓ *Roseomonas* ↓ *Roseococcus*	ZO-1 Occludin LPS	ZO-1 ↓ Occludin ↓ LPS ↑	9	7	N/A	N/A	N/A	N/A	N/A	N/A
Fu, 2022^37^; China	100 mg L^–1^ NaF	RNA extraction by Trizol reagent FQTRIM (version 0.94) was used for quality filtering and SILVA was used to compare the sequences.	Bacteroidetes Firmicutes	Bacteroidetes ↑ Firmicutes ↓ Epsilonbacteraenta ↓ Muribaculaceae ↑ *Muribaculum* ↑ *Paramuribaculum* ↑ *Dubosiella* ↑ *Lachnospriaceae_NK4A136_Group* ↓ *Lachnospriaceae* *unclassed* ↓ *Lachnoclostridium* ↓ Alistipes ↓ Parabacteroides ↓ Helicobacter ↓ *Anaerotugnum* ↓ *Ruminiclostridium* 5 ↓	IL-1β IL-6 TNF-α TLR2 NF-κB Occludin ZO-1 Claudin1 α-Defensin5 Reg3b Reg3g	IL-1β ↑ IL-6 ↑ TNF-α ↑ TLR2 ↑ NF-κB ↑ Occludin ↓ ZO-1 ↓ Claudin-1 ↓ α-Defensin5 ↓ Reg3b ↓ Reg3g ↓	N/A	N/A	N/A	N/A	N/A	N/A	N/A	N/A
Li, 2022^68^; China	200 mg kg^–1^ NaF solution	QIAamp DNA Stool Mini Kit (QIAGEN) Bacteria: 16S rDNA gene sequencing Illumina MiSeq platform FLASH was used for demultiplexing and quality filtering UPARSE software was used to cluster OTUs applying a cutoff of 97% similarity.	Firmicutes 91.9% Proteobacteria 7.6% *Enterococcus* *Aquabacterium* *Aureimonas* *Methylobacterium* *Allorhizobium, Neorhizobium, Pararhizobium, Rhizobium* *Caulobacter* *Ochrobactrum* *Vibrionimonas* *Asticcacaulis* *Enterobacter*	Firmicutes 1.2% Proteobacteria 93.2% *Enterococcus* ↓ *Aquabacterium* *Aureimonas* *Methylobacterium* *Allorhizobium* *Neorhizobium* *Pararhizobium* *Rhizobium* *Caulobacter* *Ochrobactrum* *Vibrionimonas* *Asticcacaulis* *Enterobacter*	N/A	Arginine ↑ Glutamine ↑ Adenosine ↓ Guanosine ↓ Pyrimidine metabolism ↑ Purine metabolism ↑ Arginine biosynthesis ↑ Mineral absorption ↓ Protein digestion and absorption ↓ Aminoacyl-tRNA biosynthesis ↓	217	492	N/A	N/A	N/A	N/A	N/A	N/A
Zhang, 2022^57^; China	80 mg L^–1^ NaF	Genomic DNA extraction by PowerMax DNA isolation kit Pair-end 2 × 150 bp sequencing using Illumina NovaSeq6000 platform	Proteobacteria Firmicutes to Bacteroidetes ratio Bacteroides Tenericutes Planctomycetes Firmicutes	**80 mg L^–1^ of F as NaF:** Proteobacteria 49.0% ↑ Firmicutes to Bacteroidetes ratio 255.2% ↑ Bacteroides ↓ Tenericutes ↓ Planctomycetes ↓ Firmicutes ↓	MDA SOD CAT GSH level GPx ACP LZM Muc2 ZO-1 Occludin Claudin-1	**80 mg L^–1^ of F as NaF** **30- d exposure:** MDA 96.3% ↑ SOD 30.4% ↑ CAT 40.7% ↑ GSH level 50.6% ↓ GPx 84.4% ↓ ACP 15.9% ↓ LZM 25.9% ↑ Muc2 ↓ ZO-1 **60-d exposure** Reactive oxygen species ↑ MDA ↑ ALP ↑ Myeloperoxidase ↑ Muc2 ↓ ZO-1 ↓ Occludin ↓ Claudin-1 ↑ **90-d exposure** SOD ↑ CAT ↓ GSH ↓ GPx ↓ ACP ↓ LZM ↓ Muc2 ↓ ZO-1 ↓ Occludin ↓ Claudin-1 ↓	N/A	N/A	N/A	Decreased Compared with control	N/A	N/A	N/A	Decreased Compared with control
Zhong, 2022^54^; China	NaF: 25, 50, 100, 150 mg L^–1^	Genomic DNA extraction by environmental sample DNA extraction kit (OMEGA) 16srRNA sequencing using Illumina MiSeq platform The OTUswere clustered at a similarity of 97% using parse software.	Bacteroidetes Firmicutes Proteobacteria Tenericutes Actinobacteria Elusimicrobia Verrucomicrobia *Candidatus*, Saccharibacteria Ruminococcaceae *Paenalcaligenes*	**25 mg L^–1^ NaF:** Unclassified Bdellovibrionales Ruminococcaceae Paenalcaligenes ↓ Unclassified Desulfovibrionaceae **50 mg L^–1^ NaF:** *Pelagibacterium* Unclassified Bdellovibrionales Ruminococcaceae **100 mg L^–1^ NaF:** Ruminococcaceae ↑ *Pelagibacterium* Unclassified Bdellovibrionales **150 mg L^–1^ NaF:** Pelagibacterium ↑ Unclassified Bdellovibrionales ↑ Ruminococcaceae ↓ *Roseburia* *Clostridium sensu stricto*, *Turicibacter* and *Pelagibacterium* were the same in all 3 treatments.	N/A	N/A	N/A	N/A	378.73 ± 12.30	**25 mg L^–1^ NaF: **403.88 ± 12.80 **50 mg L^–1^ NaF: **401.36 ± 21.55 **100 mg L^–1^ NaF: **381.06 ± 12.08 **150 mg L^–1^ NaF: **368.71 ± 10.42	0.94 ± 0.03	**25 mg L^–1^ NaF: **0.96 ± 0.02 **50 mg L^–1^ NaF: **0.93 ± 0.04 **100 mg L^–1^ NaF: **0.88 ± 0.05 **150 mg L–1 NaF: **0.93 ± 0.03	4.00 ± 0.29	**25 mg L^–1^ NaF: **4.26 ± 0.22 **50 mg L^–^^1^ NaF:** 3.92 ± 0.50 **100 mg L^–1^ NaF: **3.61 ± 0.37 **150 mg L^–1^ NaF: **3.86 ± 0.26
Zhu, 2022^10^; China	25, 50, and 100 mg L^–1^ F	DNA extraction using E. Z.N.A. soil DNA Kit 16srRNA sequencing by Illumina MiSeq platform OTUs were created by clustering the reads at 97% similarity using UPARSE software. (version 7.1)	Firmicutes (69.18%–85.17%) Actinobacteria (9.83%–19.58%) Bacteroidetes (1.21%–7.33%) Verrucomicrobia (0.2%– 3.07%) Lactobacillus norank-f-Erysipelotrichaceae norank-f-Bacteroidales-S24-7 Lachnospiraceae NK4A136 *Bifidobacterium*	**25 mg L^–1^ F** Actinobacteria ↓ Bacteroidetes ↓ Verrucomicrobia ↓ Firmicutes ↑ norank-f-Erysipelotrichaceae ↓ norank-f-Bacteroidales-S24-7 ↓ Lachnospiraceae NK4A136 ↓ Faecalibaculum ↓ **50 mg L^–1^ F:** Actinobacteria ↓ Bacteroidetes ↓ Verrucomicrobia ↓ Firmicutes ↑ norank-f-Erysipelotrichaceae ↓ norank-f-Bacteroidales-S24-7↓ Lachnospiraceae NK4A136 ↓ *Faecalibaculum* **100 mg L^–1^ F:** Actinobacteria ↓ Bacteroidetes ↓ Verrucomicrobia ↓ Firmicutes ↑ norank-f-Erysipelotrichaceae ↓ norank-f-Bacteroidales-S24-7 ↓ Lachnospiraceae NK4A136 ↓ *Bifidobacterium* ↓ *Faecalibaculum* ↓	Glycoproteins	**25 mg L^–1^ F:** Glycoproteins CRAMP ↑↑ β-Defensin-1↑ β-Defensin-3 ↑ **50 mg L^–1^ F:** Glycoproteins ↓ CRAMP ↑ β-Defensin-1 ↑ β-Defensin-3↑ **100 mg L^–1^ F:** Glycoproteins ↓ CRAMP ↑ β-Defensin-1 ↑ β-Defensin-3 ↑ Gene expression **25 mg L^–1^ F:** IL-17A ↑ IL-22 ↑ IL-22R IL-17RA ↓ **50 mg L^–1^ F:** IL-17A ↑ IL-22 ↑ IL-22R ↑ IL-17RA ↓ **100 mg L^–1^ F:** IL-17A ↑ IL-22 ↑ IL-22R ↑ IL-17RA ↓	6003	N/A	N/A	N/A	N/A	N/A	N/A	Decreased Compared to control
Zhang, 2023^61^; China	100 mg L^–1^ NaF Antibiotics cocktail 10 mg mL^–1^ F + antibiotic F + bacteria from feces SCFAs group F + SCFAs	RNA extraction using Trizol Western blotting ELISA 16s rRNA sequencing by Illumina NovaSeq platform	Bacteroidetes Firmicutes Verrucomicrobia *Candidatus Saccharimonas*, Muribaculaceae, *Muribaculum* Clostridia_UCG_014Odoribacter *Bacteroides*, *Lachnoclostridium* *Mycoplasma* *Rikenella* Clostridia Akkermansia *Eubacterium* *Lactobacillus* *Ruminicoccus*	Bacteroidetes ↑ Firmicutes ↓ Verrucomicrobia ↓ *Candidatus Saccharimonas* ↑ Muribaculaceae ↑ Clostridia_UCG_014 ↑ *Odoribacter* ↑ *Lachnoclostridium* ↑ *Mycoplasma* ↑ *Rikenella* ↑ Clostridia↓ Akkermansia ↓ Eubacterium ↓ *Lactobacillus* ↓ *Ruminicoccus* ↓	TLR2 Myd88 TRAF6 IKK-β TNF-α IL-1β IL-6 IFN-γ IL-10 TGF-β Acetate Propionate Butyrate	**100 mg L^–1^ NaF:** TNF-α ↑ IL-1β ↑ IL-6 ↑ IFN-γ ↑ IL-10 ↑ TGF-β ↑ TLR2 ↑ Myd88 ↑ TRAF6 ↑ IKK-β ↑ TLR4 ↑ Acetate ↓ Propionate↓ Butyrate↓	N/A	N/A	Decreased	Decreased	Decreased	Decreased	Decreased	Decreased
Zhou, 2023^76^; China	100 mg L^–1^ NaF	DNA extraction 16srRNA sequencing by Illumina MiSeq platform. LC/MS	Bacteroidetes Firmicutes Actinobacteria Proteobacteria Euryarchaeota	*Allobaculum* ↑ *Eubacterium* ↓	N/A	Pentose Glucuronate α-Ketoglutaric acid ↑	N/A	N/A	535.71 ± 65.49	491.42 ± 50.70	0.90 ± 0.033	0.90 ± 0.032	4.78 ± 0.53	4.54 ± 0.24
Tian, 2023^47^; China	100 mg L^–1^ NaF	DNA extraction 16s rRNA sequencing	Bacteroidetes Actinobacteria *Bacillus subtilis*	Bacteroidetes ↑ Actinobacteriota ↑ *B subtilis* ↓	Glutamine α-Ketoglutarate	Creatinine ↑ Beclin1 ↑ Glutamine ↓ α-Ketoglutarate↓	N/A	N/A	N/A	N/A	N/A	N/A	N/A	N/A
Huang, 2024^58^; China	24 mg kg^–1^ NaF (24 mg L^–1^)	RT-qPCR; RNA sequencing	Firmicutes, Bacteroidetes, Actinobacteria, and Proteobacteria, *Lactobacillus* (40.9 %), *Ileibacterium* (15.72%)	Firmicutes, Bacteroidetes, Actinobacteria, and Proteobacteria *Lactobacillus* (13.9 %)	Glutathione	Glutathione ↓ SLC7A11 ↑ TBARS ↑ GPX4 ↓ PTGS2 ↑ CHAC1 ↑ Rgs4 ↓	N/A	N/A	No difference between groups	No difference	No difference	No difference	No difference	No difference
Zhang XL, 2023^61^; China	100 mg L^–1^ NaF	Real-time PCR; qRT-PCR sequencing	Bacteroides *Parasutterella* *Lactobacillus*	Atoprostipes ↑ *Eubacterium* ↓	Linoleic acid metabolism, tryptophan metabolism, lipoic acid metabolism, and α-linolenic acid metabolism	Linoleic acid metabolism, tryptophan metabolism, lipoic acid metabolism, and α-linolenic acid metabolism ↑	2026	2208	2000	3000	1	1	7	7
Li D, 2023^62^; China	15 mg kg^–1^ F (15 mg L^–1^) 45 mg kg^–1^ F (45 mg L^–1^) 75 mg kg^–1^ F (75 mg L^–1^)	Real-time PCR; qRT-PCR sequencing	*Lactobacillus,* Ruminococcaceae_UCG-005, Lachnospiraceae_NK4A136_group Actinobacteria Proteobacteria Akkermansiaceae, *Akkermansia* Bacilli,* Lactobacillus*, and Lactobacillaceae	**15 mg kg^–1^ F (15 mg L^–1^ F):** *Lactobacillus* (28.73%) ↑ Ruminococcaceae_UCG-005, Lachnospiraceae_NK4A136_group ↑ Actinobacteria ↓ Proteobacteria ↓ *Verrucomicrobiae* ↑ Akkermansiaceae ↑ Akkermansia ↑ **45 mg kg^–1^ F (45 mg L^–1^ F):** Bacilli ↑ *Lactobacillus* ↑ Lactobacillaceae ↑ **75 mg kg^–1^ F (75 mg L^–1^ F):** Bifidobacteriales ↑ Bacteroidetes ↑ Actinobacteria ↑	MDA SOD GSH *NQO1* *Nrf2* *HQ1*	MDA ↓ SOD ↑ GSH ↑ *NQO1* ↓ *Nrf2* ↓ *HQ1* ↓	2240	**15 mg kg^–1^ F (15 mg L^–1^ F):** 1032 **45 mg kg^–1^ F (45 mg L^–1^ F):** 933 **75 mg kg^–1^ F (75 mg L^–1^ F):** 1293	1200	**15 mg kg^–1^ F (15 mg L^–1^ F):** 1000 **45 mg kg^–1^ F (45 mg L^–1^ F):** 800 **75 mg kg^–1^ F (75 mg L^–1^ F):** 800	N/A	N/A	7.9	**15 mg kg^–1^ F (15 mg L^–1^ F):** 7.7 **45 mg kg^–1^ F (45 mg L^–1^ F):** 7.7 **75 mg kg^–1^ F (75 mg L^–1^ F):** 7.7
Chen, 2023^63^; China	50 mg L^–1^ NaF	16S rRNA sequencing; qRT PCR	Bacteroidetes Firmicutes	Bacteroidetes↑ Firmicutes↑ Firmicutes to Bacteroidetes ratio ↑ Erysipelotrichaceae ↑ *E ramosum*↑	ZO-1 Occludin IL-1β, IL-6, TNF-α Myd88 Tlr4	ZO-1 ↓ Occludin ↓ IL-1β ↑, IL-6 ↑, TNF-α ↑ Myd88 ↑ Tlr4 ↑	440	450	450	460	1	1	6	6
Zhao, 2023^64^; China	100 mg L^–1^ NaF	16S rRNA sequencing; qRT PCR	Bacteroidota (55.824%) Firmicutes (33%) Verrucomicrobiota (5%) Proteobacteria (4%) Patescibacteria (1%) Actinobacteriota (1%) Campilobacterota (0.4%) Cyanobacteria (0.2%) Desulfobacterota (0.1%) Deferribacterota (0.02%) Firmicutes to Bacteroidota ratio (0.7%) Muribaculaceae (39%) *Alistipes* (6%) *Akkermansia* (5%) *Rikenella* (3%) *Blautia* (3%) *Turicibacter* (3%) *Bacteroides* (2%) *Odoribacter* (2%) Clostridia_UCG-014 (2%) *Alloprevotella* (2%)	Bacteroidota (70.8%) ↑ Firmicutes (23%) ↓ Verrucomicrobiota (0.02%) ↓ Proteobacteria (3.5%) ↓ Patescibacteria (1.5%) ↑ Actinobacteriota (0.7%) Campilobacterota (0.5%) ↑ Cyanobacteria (0.02%) ↓ Desulfobacterota (0.1%) ↓ Deferribacterota (0.003%) ↓ Firmicutes to Bacteroidota ratio (0.3%) ↓ Muribaculaceae (47%) ↑ *Alistipes* (4%) ↓ *Akkermansia* (0.02%) ↓ *Rikenella* (4%) ↑ *Blautia* (0.1%) ↓ *Turicibacter* (0.3%) ↓ *Bacteroides* (4%) ↑ *Odoribacter* (4%) ↑ Clostridia_UCG-014 (4%) ↑ *Alloprevotella* (2%)	BDNF-PI3K/AKT PI3K Bak Bax Caspase-7 Protein Caspase-3	BDNF-PI3K/AKT ↓ PI3K ↑ Bak ↑ Bax ↑ Caspase-7 ↑ Protein Caspase-3 ↑	N/A	N/A	N/A	N/A	N/A	N/A	N/A	N/A
Mo Z, 2023^65^; China	25, 50, 100, and 150 mg L^–1^ NaF	16S rRNA sequencing	Bacteroidetes (81%) Firmicutes (17%) Actinobacteria (0.3%) Proteobacteria (0.3%) Ruminococcaceae *Uricibacter* Lachnospiraceae *Roseburia* *Clostridium sensu stricto*	**25 mg L^–1^ NaF:** Bacteroidetes (78%) ↓ Firmicutes (20%) ↑ Actinobacteria (0.4%) ↑ Proteobacteria (0.3%) *Uricibacter* ↑ Lachnospiraceae ↑ *Roseburia* ↑ *C sensu stricto* ↑ **50 mg L^–1^ NaF:** Bacteroidetes (76%) ↓ Firmicutes (23%) ↑ Actinobacteria (0.2%) ↓ Proteobacteria (0.2%) ↓ *Uricibacter* ↓ Lachnospiraceae ↓ *Roseburia* ↓ *C sensu stricto* ↓ **100 mg L^–1^ NaF:** Bacteroidetes (86%) ↑ Firmicutes (13%) ↓ Actinobacteria (0.4%) ↑ Proteobacteria (0.2%) ↓ *Uricibacter* ↓ Lachnospiraceae ↓ *Roseburia* ↓ *C sensu stricto* ↓ **150 mg L^–1^ NaF:** Bacteroidetes (81%) Firmicutes (18%) ↑ Actinobacteria (0.5%) ↑ Proteobacteria (0.3%) Ruminococcaceae ↑ Uricibacter↓ Lachnospiraceae ↓ *Roseburia* ↓ *C sensu stricto* ↓	d-lyxose ketol-isomerase Alanine-synthesizing transaminase Fis family transcriptional regulator CoA-dependent NAD(P)H sulfur oxidoreductase 3D-(3,5/4)-trihydroxycyclohexane-1,2-dione acylhydro-lase (decyclizing)	**25 mg L^–1^ NaF:** d-lyxose ketol-isomerase ↑ Alanine-synthesizing transaminase ↑ Fis family transcriptional regulator ↑ CoA-dependent NAD(P)H sulfur oxidoreductase ↑ 3D-(3,5/4)-trihydroxycyclohexane-1,2-dione acylhydro-lase (decyclizing) ↑ **50 mg L^–1^ NaF:** d-lyxose ketol-isomerase ↑ Alanine-synthesizing transaminase ↑ Fis family transcriptional regulator ↑ CoA-dependent NAD(P)H sulfur oxidoreductase ↑ 3D-(3,5/4)-trihydroxycyclohexane-1,2-dione acylhydro-lase (decyclizing) ↑ **100 mg L^–1^ NaF:** d-lyxose ketol-isomerase,↑ Alanine-synthesizing transaminase ↑ Fis family transcriptional regulator ↑ CoA-dependent NAD(P)H sulfur oxidoreductase ↑ 3D-(3,5/4)-trihydroxycyclohexane-1,2-dione acylhydro-lase (decyclizing) ↑ **150 mg L^–1^ NaF:** d-lyxose ketol-isomerase ↑ Alanine-synthesizing transaminase ↑ Fis family transcriptional regulator ↑ CoA-dependent NAD(P)H sulfur oxidoreductase ↑ 3D-(3,5/4)-trihydroxycyclohexane-1,2-dione acylhydro-lase (decyclizing) ↑	N/A	N/A	N/A	N/A	N/A	N/A	N/A	N/A
Wu, 2024^59^; China	100 mg L^–1^ NaF	Real-time PCR; qRT-PCR sequencing	Researchers looked for supportive effects of *Bifidobacterium* to relieve the liver and ileum damage done by F. Microbial data N/A	Researchers looked for supportive effects of *Bifidobacterium* to relieve the liver and ileum damage done by F. Microbial data N/A	IL-1β, IL-6, TNF-α ASBT, IBABP, OST-α, and OST-β	IL-1β ↑, IL-6 ↑, TNF-α ↑	N/A	N/A	N/A	N/A	N/A	N/A	N/A	N/A
Zhao, 2024^60^; China	200 mg L^–1^ NaF	Real-time PCR; qRT-PCR sequencing	Firmicutes, Bacteroidetes *Lactobacillus* *L. vaginalis*	Bacteroidetes ↓ *Lactobacillus* ↑ *L vaginalis* ↓ Firmicutes ↑	IL-1β, IL-6, TNF-α Occludin *mucin-2* mRNA	IL-1β ↑, IL-6 ↑, TNF-α ↑ Occludin ↑ *mucin-2* mRNA ↑ GPRCs ↓	N/A	N/A	N/A	N/A	N/A	N/A	N/A	N/A
Feng, 2024^67^; China	0.57 and 5.7 mg L^–1^ F35	RT-qPCR 16S rRNA sequencing	Bacteroidetes Firmicutes *Lactobacillus*	**0.57 mg L^–1^ F35 and 5.7 mg L^–1^ F35** Bacteroidetes↑ Firmicutes *Lactobacillus* ↓	IL-1β TNF-α IL-10 TLR4 *NF-κB* ZO-1 Occludin	**0.57 mg L^–1^ F35** IL-1β TNF-α IL-10 ↓ TLR4, ↑ *NF-κB* ↑ **5.7 mg L^–1^ F35:** IL-1β ↑ TNF-α ↑ IL-10 ↓ TLR4 ↑ *NF-κB* ↑ SOD ↓ CAT ↓ GSH ↓ MDA ↑ AKT ↓ PI3K ↓ ZO-1 ↓ Occludin↓	458	151	No difference between the groups	No difference	No difference	No difference	No difference	No difference
**Human studies**														
Chen, 2021^14^; China	1, 2, 10, and 15 mg L^–1^ F	DNA extraction by TIANGEN Biotech (Beijing, China) Illumina sequencing Mothur evaluated α-diversities. R package evaluated β-diversities OTUs were taxonomically classified using UCLUST.	Proteobacteria Fusobacteria Firmicutes Bacteroidetes Actinobacteria	**1 mg L^–1^ F:** Proteobacteria ↓ Fusobacteria ↑ **2 mg L^–1^ F:** Proteobacteria ↓ Fusobacteria ↑ **10 mg L^–1^ F:** Proteobacteria ↑ Fusobacteria ↓ **15 mg L^–1^ F:** Proteobacteria ↑ Fusobacteria ↓ Lactobacilli decrease in a dose-dependent manner	Acetic acid Propionic acid Butyric acid	Acetic acid Propionic acid Butyric acid At 1 and 2 mg L^–1^ F no effect on KEGG pathway, but higher concentrations changed the functional modules.	3	**1 mg L^–1^ F:** 5 **2 mg L^–1^ F:** 2 **10 mg L^–1^ F:** 1 **15 mg L^–1^ F:** 9	N/A	N/A	N/A	N/A	N/A	N/A
Zhou, 2023^76^; China	N/A	DNA extraction 16s rRNA sequencing by Illumina MiSeq platform LC/MS	Bacteroidetes Firmicutes Actinobacteria Proteobacteria Tenericutes Paraprevotellaceae *Paraprevotella* Leuconostocaceae	Acidobacteria Paraprevotellaceae ↑ *Paraprevotella* ↑ Leuconostocaceae ↑	Pentose Glucuronate	Pentose ↑ Glucuronate ↑	155	318	681.87 ± 139.09	653.49 ± 153.10	0.95 ± 0.03	0.94 ± 0.03	6.03 ± 0.53	5.88 ± 0.51
Yoon, 2019^74^; Korea	^18^F-FDG intestinal uptake; no dose mentioned	16s rRNA sequencing	N/A; this was a cohort study of patients with breast cancer. Authors looked at, when performing the scan, how much intestinal uptake is there of ^18^F-FDG.	**Higher uptake:** *Enterobacter* ↑ Ruminococcaceae ↓ **Lower uptake:** *Enterobacter* ↓ Ruminococcaceae ↑	N/A; this was a cohort study of patients with breast cancer. Authors looked at, when performing the scan, how much the intestinal uptake is there of ^18^F-FDG.	**Higher uptake:** TNF-α ↑ IL-1 **Lower uptake:** TNF-α ↓ IL-1	N/A	**Higher uptake:** 1160 **Lower uptake:** 1092	N/A	**Higher uptake:** 0.8 **Lower uptake:** 0.8	N/A	**Higher uptake** **Lower uptake**	N/A	**Higher uptake:** 5.46 ± 0.71 **Lower uptake:** 5.46 ± 0.71
Wang, 2023^75^; China	N/A	DNA extraction 16s rRNA sequencing by Illumina NOVASeq platform	Bacteroidetes Firmicutes Proteobacteria	Bacteroidetes ↓ Firmicutes ↓ Proteobacteria ↑ Acidobacteriota ↑, Fusobacteriota ↑ Desulphobacterota ↑ *Prevotella* ↓ Bacteroides ↓ *Escherichia-Shigella* ↓ *Faecalibacterium* ↓ *Bifidobacterium* ↓ *Streptococcus* ↓	5-Hydroxyindoleacetic acid Tryptamine Indole acetaldehyde	5-Hydroxyindoleacetic acid ↓ Tryptamine ↓ Indole acetaldehyde ↓	13 454	7742	N/A	N/A	N/A	N/A	N/A	N/A

a The groups without an increase or decrease signs under gene expressions and metabolites indicate that either the expression was found only in 1 group or no significant difference was found between the 2 groups.

b Control group: without fluoride exposure.

c Intervention group: those given fluoride as a treatment or intervention.

Abbreviations: ^18^F-FDG, fluorine-18 fluorodeoxyglucose; ACP, acid phosphatase; ALP, alkaline phosphatase; ASBT, apical sodium-dependent bile acid transporter gene; Bax, BCL2-associated X protein; BCL, B-cell lymphoma; BDNF, brain derived neurotrophic factor; CAT, catalase; CRAMP, cathelicidin-related antimicrobial peptide; CREB, cAMP response elements binding protein; cyt, cytochrome; DAO, diamine oxidase; F, fluoride; F35, polyfluorinated ether sulphonate; FFAR2, free fatty acid receptor 2; FSH, follicle-stimulating hormone; GPCR, G protein couple receptor; GPX, glutathione peroxidase; GSH, glutathione; IBABP, Ileal bile acid binding protein; IKK-β, inhibitor of nuclear factor κ-B kinase subunit β; IL, interleukin (cytokine); KEGG, Kyoto Encyclopedia of Genes and Genomes; LC/MS, liquid chromatography mass spectrometry; LC3, microtubule-associated protein light chain 3; LPS, lipopolysaccharide; LZM, lysozyme; MAG, myelin-associated glycoprotein; MBP, myelin basic protein; MDA, malondialdehyde; MOG, myelin oligodendrocyte glycoprotein; MUC2, mucin glycoprotein-2; N/A not applicable/not mentioned; NaF sodium fluoride; NCAM, neural cell adhesion molecule; NQO1, quinone dehydrogenase 1; OTU, operational taxonomic unit; p62, ubiquitin-binding protein; PFOA, perfluorooctanoic acid; PLP, pyridoxal phosphate; PTGS, Prostaglandin-endoperoxide synthase; qPCR, quantitative polymerase chain reaction; RT-PCR, real-time–polymerase chain reaction; SCF, stem cell factor; SCFA, short-chain fatty acid; scRNA-seq, single-cell RNA sequencing; SDS-PAGE, Sodium dodecyl-sulfate polyacrylamide gel electrophoresis; SOD, superoxide dismutase; T-AOC, Total Antioxidant Capacity; TBARS, thiobarbituric acid reactive substances; TGF, transforming growth factor; TLR, toll-like receptor; TNF, tumor necrosis factor; TRAF, tumor necrosis factor receptor–associated factor; ZO, Zonula occludens; ↑, increase; ↓, decrease.

The total sample size across all the included animal studies was 3249, of which at least 45% were male and 19% were female; 12 (29%) studies were unable to report the sex of participants. The 4 included human studies reported a total sample size of 217 participants, with 2 studies indicating participants’ sex (Table 2).

### Intervention Characteristics

Most of the included studies (*n* = 35; 73%) used NaF as a source of fluoride in water or diet as an intervention; 3 studies used perfluoroalkyl fluorides (organic fluoride; ie, sodium fluoroacetate^44^^,^^69^ and perfluorooctanoic acid^66^); and 1 study looked at the effect of polyfluorinated ether sulphonate on the gut microbiota of mice.^67^ The in vivo NaF doses used in water and diet in the animal studies were 0.5,^49^ 5,^49^ 24,^58^ 25,^10^^,^^54^^,^^65^ 50,^10^^,^^48^^,^^49^^,^^54^^,^^63^^,^^65^ 80,^55^^,^^57^ 100,^10^^,^^31–33^^,^^35–37^^,^^40^^,^^45–48^^,^^53–55^^,^^57^^,^^59^^,^^62^^,^^64^^,^^67^^,^^65^^,^^76^ 150,^54^^,^^65^ 200,^31^^,^^35^^,^^39^^,^^60^^,^^68^ 300,^35^ 400,^35^^,^^41–43^ 500,^35^ 750,^41^, 800,^41^ and 1200 mg L^–1^.^41–43^ Three studies indicated only fluoride levels and used 4, 10, 15, 45, 50, and 75 mg L^–1^ fluoride.^34^^,^^53^^,^^62^ Three more studies used 0.5, 1, and 3 mg kg^–1^ perfluorooctanoic acid^66^; 0.57 and 5.7 mg L^–1^ polyfluorinated ether sulphonate^67^; and 0.2 mg kg^–1^ sodium fluoroacetate.^44^ The in vitro doses used were 0.1,^71^ 1,^71^ 4.76,^70^ 10,^71–73^ 20,^72^^,^^73^ 30,^72^^,^^73^ 40,^72^^,^^73^ 50,^72^^,^^73^ and 100^71^ mM NaF and 20 mM sodium fluoroacetate.^69^ The ex vivo study of human fecal samples used 1, 2, 10, and 15 mg L^–1^ F.^14^ The details of doses are listed in Table S4.

Most in vivo animal studies (83%) reported the long-term association (1-6 months) between fluoride exposure and gut microbiome composition; 19% of animal and 1 human study reported short-term outcomes (1 day). Two animal studies compared participants at baseline before the inception of any intervention (ie, pre-post studies).^53^^,^^69^

Results from all the 49 studies are presented narratively because they were unable to be statistically pooled.

### Quality Assessment

The methodological quality of the included studies was assessed based on the Mixed Methods Appraisal Tool using criteria specific to observational studies (including cohort studies and case-control studies), quantitative studies (including randomized and nonrandomized controlled studies), and laboratory-based studies. In this systematic review, the included studies were mainly of high quality (79% of studies). As shown in Figure S1, 39 studies (79%)^10^^,^^14^^,^^31–43^^,^^45^^,^^46^^,^^49–51^^,^^54–56^^,^^58–67^^,^^70^^,^^71^^,^^74^^,^^76^ met all the quality assessment criteria, and 4 studies (8%)^52^^,^^57^^,^^68^^,^^72^ met 7 of 8 assessment criteria. Three studies (6%)^44^^,^^53^^,^^73^ met 6 criteria, and 3 studies (6%) met 5 assessment criteria.^58^^,^^69^^,^^75^ The risk of selection bias remained low because most of the studies were deemed of good quality.

### Effect of Fluoride on Gut Microbiota (Primary Outcome)

#### Shifts in Microbial Diversity/Richness (Alpha and Beta Diversity)

An increase or decrease in alpha diversity indices,^77^ with Chao 1 used to estimate the number of species in a community and the Shannon and Simpson indices used to estimate the abundance of each species,^28^^,^^78^ in fluoride-treated groups when compared with control groups indicates fluoride is affecting the microbial community structure. Alpha diversity differences were estimated in 17 of the included studies (35%) and computed using the aforementioned 3 diversity indices.^10^^,^^31^^,^^38^^,^^39^^,^^43^^,^^45^^,^^50^^,^^52^^,^^54^^,^^57^^,^^58^^,^^61–63^^,^^67^^,^^74^^,^^76^  Table 3 and Table S5 show an increase and decrease in Chao 1, Simpson, and Shannon diversity indices at different doses and durations of fluoride. (Table 3 and Table S5). Eight of the included animal studies^10^^,^^38^^,^^45^^,^^54^^,^^56^^,^^57^^,^^62^^,^^76^ reported a decrease in alpha diversity and richness (by all 3 indices) after exposure to high doses of fluoride (ie, 50, 80, 100, 150, and 750 mg L^–1^ NaF in both water and diet) compared with the control group for 1-4 months. Conversely, some studies^31^^,^^39^^,^^43^^,^^50^^,^^52^^,^^54^^,^^61^^,^^76^ reported increased alpha diversity and richness at 100, 200, 400, and 1200 mg L^–1^ NaF in water and diet for 16 hours to 2 months. Two studies found no significant differences in alpha diversity.^58^^,^^67^ Zhong et al^54^ and Zhu et al^10^ found that microbial diversity and richness were negatively correlated with fluoride dose.

Nine studies^10^^,^^14^^,^^34^^,^^36^^,^^37^^,^^54^^,^^55^^,^^57^^,^^76^ also assessed the beta diversity differences between control and treated groups by unconstrained principal coordinate analysis. Of these, 2 studies^14^^,^^34^ indicated no differences in beta diversity after the administration of fluoride compared with a control. In the remaining studies, the numeric data were not available to be displayed in tables. Four good-quality studies^10^^,^^54^^,^^55^^,^^57^ indicated large differences in beta diversity after fluoride exposure. Beta diversity estimates the similarity and dissimilarity (uniqueness) between the populations (samples).^79^

#### Fluoride and Abundance of Gut Microbial Species

The abundance of gut microbiota was estimated and compared between the control and interventional groups in all included studies. Data were analyzed by fluoride dose and duration.

##### Effects of fluoride on the abundance of human gut microbiota

Limited studies (*n* = 4) conducted with human participants considered the impact of ingested fluoride on gut microbiota. One of the ex vivo studies of human feces, which used a fermenter, indicated a low dose (1-2 mg L^–1^) of NaF did not show a significant difference in the abundance at phylum and genus levels but may promote taxa associated with health, including *Faecalibacterium* and *Lactobacillus.* However, high doses (10-15 mg L^–1^ NaF) significantly increased the relative abundance of Proteobacteria (synonym Proteobacteriota) (Table 3).^14^ Two population studies explored the correlation between dental fluorosis and gut microbiota change by analyzing the fecal samples of children and adult patients with fluorosis. The patients had significant variations in microbiota composition and abundance compared with healthy participants. This was characterized by an increase in the relative abundance of Verrucomicrobiota*,* Desulfobacterota, Acidobacteriota, and Proteobacteria, and a significant decline in the relative abundance of Firmicutes and Bacteroidetes at the phylum level. At the genus level, the abundance of *Bifidobacterium*, and *Faecalibacterium* was reduced significantly.^75^^,^^76^ In another case-control study, the effect of fluorine-18 fluorodeoxyglucose (^18^F-FDG) positron emission tomography on the gut microbiota of patients with breast cancer was investigated. The researchers used ^18^F-FDG to determine the breast cancer stage and found a negative correlation between intestinal ^18^F-FDG uptake and the abundance of members of the Ruminococcaceae, which lead to mucosal inflammation in patients due to the increase of proinflammatory cytokines.^74^

The results from all the human studies suggest high fluoride exposure changed the taxonomic composition of gut microbiota in humans by disturbing the balance between useful microorganisms (namely, Ruminococcaceae, Firmicutes, *Faecalibacterium*, *Lactobacillus*, and Bacteroidetes) and pathogenic microorganisms (eg, Proteobacteria, Acidobacteriota). This disturbance can lead to IBD, colitis, and even cancers, due to lack of microbial metabolites (eg, SCFAs) that control the metabolic pathways. Moreover, the low dose can stimulate the growth of beneficial bacteria. However, the population studies did not mention the dose that was responsible for fluorosis.^74–76^

##### Chronic and acute effects of fluoride on abundance change patterns at phylum and genus levels in animal models

Overall, data were classified by different doses and duration of fluoride. Across animal species, we found consistent exposure to high doses of fluoride may result in disturbance in gut microflora. However, the phyla or genera most affected differ between different animal types.

##### Effect of fluoride on gut microbiota abundance and composition in rodents

In rodents, most studies (72%) reported a dramatic decrease in the abundance of Firmicutes at the phylum level and lactobacilli at the genus level upon exposure to a high dose of fluoride (25-100 mg L^–1^ NaF) compared with a control.^31^^,^^36^^,^^37^^,^^46^^,^^50–52^^,^^56^^,^^75^ Similarly, the abundance of Bacteroidetes could also be affected by fluoride exposure. Among the rat and mice studies, 10 reported an increase in Bacteroidetes level and a decrease in Parabacteroides and Bacteroidales at 100 mg L^–1^ NaF.^31^^,^^36^^,^^49–52^^,^^56^

Of the Actinobacteria*, Bifidobacterium* was also influenced by fluoride intervention, with an increased abundance indicated in 5 studies^31^^,^^47^^,^^50^^,^^51^^,^^75^ and a decrease in 3 studies when animals were exposed to 50-200 mg L^–1^ NaF.^36^^,^^49^^,^^59^

Rarer gut phyla, such as Proteroidetes,^49^ Cyanobacteria,^50^ Proteobacteria,^31^ Atoprostipes, Lachnospiraceae,^61^ Erysipelotrichaceae,^63^ and Tenericutes (synonym Tenericuteota)^36^ increased upon high fluoride (25-150 mg L^–1^ NaF) exposure, but populations of Saccharibacteria^31^^,^^49^ and Verrucomicrobia (synonym Verrucomicrobiota)^36^^,^^49^^,^^56^ decreased. Four studies investigated the dose-response (low to high dose) effects of fluoride on the gut microbiota of mice. Three of the studies used doses of 25, 50, 100, and 150 mg L^–1^ NaF for 70 days^10^ and 12 weeks.^54^^,^^65^ Fluoride led to significantly lower levels of Actinobacteria, Bacteroidetes, and Verrucomicrobia and significantly higher levels of Firmicutes, whereas *Turicibacter*, Lachnospiraceae, *Roseburia*, and *Clostridium sensu stricto* were negatively associated with fluoride exposure. Ruminococcaceae populations increase with increasing fluoride exposure doses.^10^^,^^54^^,^^65^ On the contrary, 1 study used 15, 45, and 75 mg L^–1^ NaF for 3 months and found that low fluoride exposure upregulated the abundance of *Lactobacillus* and Lachnospiraceae*;* bacilli*, Lactobacillus*, and Lactobacillaceae were also dominant in the 45 mg L^–1^ NaF group; and Bifidobacteriales, Bacteroidetes, and Actinobacteria were dominant taxa in the 75 mg L^–1^ NaF group.^62^

However, in 1 study of mice, by Yasuda et al,^53^ applicable fluoride concentrations of 4 mg L^–1^ and 4 mg L^–1^ plus gavage resulted in the treated groups having a reduction in acidogenic oral bacteria, but there was no impact on the gut microbiome. The researchers concluded that most of the fluoride may be absorbed in the upper part of the gastrointestinal tract.^53^

Two studies observed the effect of perfluoroalkyl compounds on mice gut microbiota.^66^^,^^67^ Shi et al^66^ detected that exposure to perfluorooctanoic acid increased Bacteroidetes and decreased Firmicutes populations significantly. At the genus level, exposure to perfluorooctanoic acid decreased the abundance of *Bifidobacterium* and *Ruminococcus*.^66^ Feng et al^67^ found that 5.7 mg L^–1^ polyfluorinated ether sulphonate decreased the abundance of lactobacilli but increased that of *Staphylococcus*.^67^

High doses of NaF (range, 25-200 mg L^–1^) with long-term exposure (1-6 months) most often were administered in studies using rat models, and these demonstrated that excessive fluoride exposure impacts gut microbiota composition, although variations in specific phyla and genera abundances were observed in different studies using the same dose and duration of fluoride.

###### Impacts of fluoride dose and duration on the microbial abundance in other animal models

In silkworm^39^^,^^68^^,^^70^ and *Bufo gargarizans* tadpole^49^ animal models, increased exposure to a high dose of fluoride (200 mg L^–1^ and 5-50 mg L^–1^ NaF, respectively) led to a decrease in *Enterococcus* and *Bacillus* in silkworms and Firmicutes, Synergistetes (synonym Synergistota), and *Bacteroides* in *Bufo gargarizans* but an increase in *Staphylococcus* in silkworm and in Deltaproteobacteria*,* Endomicrobia, and Spirochaetes (synonym Spirochaetota) in *B gargarizans*.

Studies of bird models such as laying hens, broiler chickens, and ducks usually involved high concentrations of fluoride (400-1200 mg kg^–1^). Findings showed that at high doses of fluoride (given as NaF, 1200 mg kg^–1^ and 800 mg kg^–1^ in diet) the abundance of *Lactobacillus* and *Bifidobacterium* was lower and that of *Escherichia-Shigella,* Gammaproteobacteria*,* Enterobacteriaceae, *Streptococcus*, and *Staphylococcus* was higher compared with controls after both short- (16 hours) and long-term (21-59 days) exposure.^42^^,^^43^ On the contrary, in ducks, an increased richness was observed for Bacteroidetes and Firmicutes, but lower richness for Verrucomicrobia and Proteobacteria upon intake of 750 mg kg^–1^ NaF for 28 days.^38^

Fish models (common carp, zebrafish)^55^^,^^57^ indicated that exposure to high doses of fluoride (ie, 80 mg L^–1^ NaF for 90 days) can affect the abundance of Fusobacteria (synonym Fusobacteriota), Firmicutes, Verrucomicrobia, Proteobacteria, and Actinobacteria.

Overall, when comparing specific bacterial phyla and genera within the gut microbiota of animals treated with fluoride relative to the control, the most consistent findings were a lower abundance of Firmicutes and Verrucomicrobia^31^^,^^35^^,^^42–44^^,^^46^^,^^47^^,^^49^^,^^50^ followed by an increase in abundance of Bacteroidetes, Actinobacteria, *Streptococcus*, *Staphylococcus,* Desulfobacterota, and Proteobacteria^31^^,^^36^^,^^42^^,^^49^^,^^51^^,^^52^^,^^75^^,^^76^ in the populations receiving high doses of fluoride (eg, 50-1200 mg L^–1^) in the form of NaF. Some studies also showed an increase in the abundance of Firmicutes and a decrease in Bacteroidetes.^10^^,^^32^^,^^37^ Many genera were implicated across the control and treated groups, including the lower abundance of lactobacilli, *Faecalibacterium*, and *Bifidobacterium*^31^^,^^32^^,^^36^^,^^37^^,^^40^^,^^46^^,^^49^^,^^50^^,^^52^ (important in maintaining intestinal function), as well as higher abundance of *E coli*.^42^ On the other hand, the included articles in this review also indicated that low fluoride doses (0.5-25 mg L^–1^ NaF) might upregulate the abundance of probiotics (eg, *Lactobacillus*, Lachnospiraceae, bacilli, Lactobacillaceae) in animals.

#### In Vitro Models

All 3 in vitro studies used fluoride concentrations of 0.1-100 mM NaF. Findings indicated a biphasic response with fluoride-inducing bacterium growth at a low dose (0.1 mM NaF)^71^ and a dramatic increase in bacterium growth and enzyme production, especially of enolase. However, the growth of the microorganisms, especially lactobacilli, was inhibited as the dose increased from 10 mM to 100 mM NaF (Table 3).^71–73^

### Secondary Outcomes

#### Effect of Fluoride Dose and Duration on Microbiota-Associated Functions (Transcriptomic and Metabolomic Profile)

##### Human studies

The ex vivo human study observed metabolite concentrations after fluoride exposure. Both low (1-2 mg L^–1^ NaF) and high doses (10-15 mg L^–1^ NaF) of fluoride had no significant effects on the production of SCFAs^14^ (Table 3 and Table S6). The population-based studies revealed the depletion of the relative absence of functional genes associated with pentose and glucuronate interconversion (a step-in carbohydrate metabolism) in children with fluorosis. These findings were correlated with the increase in Acidobacteriota.^76^ Acidobacteriota contain the genes that can degrade polysaccharides and are involved in pentose and glucuronate interconversion (carbohydrate metabolism). An abnormal increase or decrease in the expression of these genes can result in several diseases, such as familial tumoral calcinosis.^80^^,^^81^ Additionally, 3 tryptophan metabolites (namely, 5-hydroxy indole acetic acid, tryptamine, and indole acetaldehyde) that are directly produced by the gut microbiota were significantly decreased in adult patients with fluorosis. This decrease was correlated with the decreased ratio of Firmicutes to Bacteroidetes and increased abundance of Proteobacteria, Acidobacteriota, and Verrucomicrobiota.^75^ Tryptophan metabolites are important for regulating host physiology and an aberrant decrease in their production is associated with IBD and colorectal cancer.^82^

##### Animal studies

In vivo studies showed that fluoride intake by rodents of 100-150 mg L^–1^ NaF for 1-6 months could lead to a decrease in levels of SCFAs^10^; digestive enzymes such as amylase, trypsin, and lipase^46^; antioxidative enzymes such as superoxide dismutases (SODs); metalloenzymes such as catalase^32^; glycoproteins^31^^,^^49^; and p62 proteins.^40^ Fluoride could also affect hormones, and exposure to fluoride resulted in a decrease in glutathione (*GSH*), follicle-stimulating hormone, and gastrotropin levels.^34^^,^^40^^,^^46^ Fluoride supplementation stimulated the production of proinflammatory cytokines interleukin *(IL)-17A, IL-22, IL-1β, IL-6*, tumor necrosis factor-α, and *NF-κB*,^10^^,^^37^^,^^56^ malondialdehyde (*MDA*) levels, and secretory immunoglobulin A (SIgA) antibody levels,^32^^,^^46^ which could be due to the disturbance in the Firmicutes to Bacteroidetes ratio (Table 3 and Table S6). However, the low dose of fluoride (15 and 45 mg L^–1^ NaF; 0.57 mg L^–1^ F35) decreased *MDA* and elevated *SOD* and *GSH* levels.^62^^,^^67^

Similarly, a significant upregulation of mRNA expression of pro-inflammatory cytokines cathelin-related antimicrobial peptide (*CRAMP*), *β-defensin-1, β-defensin-3*, and light chain 3 was observed at high doses (80-100 mg L^–1^ NaF) and long exposure of up to 70 days.^10^^,^^37^^,^^40^^,^^56^ A sharp decrease in mRNA expression levels of brain-derived neurotrophic factor (*BDNF*), cAMP-responsive element, stem cell factor, *Bclxl*, occludin, Zonula occludens-1, and claudin was observed under similar conditions.^37^^,^^50^^,^^63^^,^^64^^,^^66^ However, a low fluoride dose of 15 mg L^–1^ reduced the expression of *NQO1* and **Nrf2** genes, which are associated with the progression of the cell cycle and triggering immune responses, respectively.

Silkworm^39^^,^^68^ and bird^42^^,^^43^ models also showed a similar effect with a reduction in energy metabolic enzymes and SCFAs, respectively, at higher fluoride doses (200, 750, and 1200 mg L^–1^ NaF), but the production of proinflammatory cytokines was accelerated (Table 3 and Table S6). Silkworms exposed to 200 mg L^–1^ NaF had upregulated expression levels of antimicrobial peptides *Att2, CecA,* Beclin 1, *Atg5, Atg7, p62*, and *Lys*, and downregulated expression levels of *CecB6, CecD*, and *Leb1*were observed after 36 hours.^47^^,^^68^ However, 4.76 Mm NaF inhibited E11 enzyme family genes and energy-synthesizing genes.^70^ Birds exposed to 1200 and 400 mg L^–1^ NaF for 59 days had downregulated expression levels of *SIgA*, mucin 2 (*MUC2*), Zonula occludens-2, and claudin-4.^42^^,^^43^

In fish models, a study of common carp focused on the effect of fluoride (80 mg L^–1^ NaF) for 90 days on tight junction protein expression. The authors reported a significant decrease in Zonula occludens-1 and occludin levels.^55^ In zebrafish, the effects of the same dose were observed on immune-related enzymes but at different time points. After exposure for 30 days, there was an increase in the levels of *MDA, SOD*, and catalase but a decrease in *GSH*. After 60- and 90-day exposure, levels of reactive oxygen species, *MDA*, and myeloperoxidase were enhanced. However, the levels of catalase, *GSH*, acid phosphatase, and lysozyme were lowered and were associated with numbers of Firmicutes^54^ (Table 3 and Table S6).

## Discussion

Fluoride from food and water is initially absorbed in the stomach (30%) and converted into hydrogen fluoride. The pharmacokinetics of fluoride are largely governed by the pH of the stomach. Its absorption is reduced as the pH increases.^24^^,^^83^ Hydrogen fluoride can easily diffuse across the cell membrane and gastric epithelium as fluoride ions and enter the gastrointestinal tract. Fluoride is almost completely and rapidly absorbed in the gastrointestinal tract harboring the gut microbiome, with a half-life of about 30 minutes. The gastrointestinal tract bacteria secrete acids and enzymes that assist in fluoride absorption.^84^^,^^85^ The absorbed fluoride is transported via the blood to various organs of the body. The excess fluoride is excreted via urine. Unabsorbed fluoride in the gut is excreted through feces.^84^^,^^85^

This systematic review shows the possible relationship between fluoride and gut microbiota composition and its associated outcomes at a concentration of ≥ 50 mg L^–1^ NaF in animals for ≥ 1 month and ≥ 10 mg L^–1^ NaF in humans for 24 hours. However, the studies included were substantially heterogeneous, with different model animals, biomarkers, and methods used to estimate gut microbial composition. Overall, all studies concluded that fluoride in suitable amounts (per the WHO recommendation for each species type) boosts gut microbial growth (probiotics) but, in excess, discourages microbial growth.

The most relevant changes affecting the main phyla of the intestinal microbial community after fluoride exposure include Firmicutes (Firmicuteota), Actinobacteria (Actinobacteriota) and Bacteroidetes (Bacteroidota), with either a decrease or an increase in relative abundance.

### Shifts in alpha and beta diversity in gut microbiota

A variation in alpha and beta diversity in the fluoride group compared with the control group indicated that fluoride exposure posed a prominent influence on gut microbiota. However, the effect patterns in different studies varied, which might be due to different exposure levels of fluoride, biomarkers used, and the use of diverse animal models with different genetic makeup, sensitivity, and other characteristics such as age, sex, and weight. The differences in the alpha and beta diversities between the control and intervention groups indicated high doses of fluoride could affect species richness. Although the studies do not provide information on changes in the abundance of specific taxa, they give us access to a broader change or difference in the composition of microorganisms. Positive or negative health implications associated with changes in diversity depend on which species are present abundantly and which are suppressed.

### Response of specific microbial members to Fluoride in human and animal studies

The majority of human and animal studies showed the effects of fluoride on Firmicutes (synonym Firmicuteota), Bacteroidetes (synonym Bacteroidota) and Actinobacteria (synonym Actinobacteriota). Associated genera were also implicated across the control and treated groups, including the lower abundance of Lactobacilli, *Faecalibacterium*, and *Bifidobacterium*^31^^,^^32^^,^^36^^,^^37^^,^^40^^,^^46^^,^^50^^,^^52^ (important in maintaining intestinal function), as well as higher abundance of *E coli*.^42^ Lactobacilli reinforce the intestinal barrier while inhibiting pathogens’ growth by producing metabolites.^86^  *Bifidobacterium* and *Faecalibacterium* maintain the immune system and help maintain T-helper cell and regulatory T-cell balance; a disturbance leads to changes in cellular expression, a potential cause of intestinal inflammation and associated disorders.^87^ Verrucomicrobia include intestinal mucosal bacteria that promote intestinal health and glucose homeostasis and play a role at an interface between the human gut microbiome and host tissues.^88^ Actinobacteria can survive in toxic environments. The opportunistic pathogens of Proteobacteria can cause a major structural imbalance of gut microbiota. At the same time, fusobacteria have been widely recognized as potential inducers of T regulatory cells or carcinogens promoting autophagic activation.^88^ Desulfobacterota are sulphur-reducing bacteria associated with the activation of immune response and inflammation. They can degrade butyrate (an SCFA), which inhibits inflammation. The abrupt increase or decrease in the abundance of members of the Firmcuteota, Verrucomicrobiota Actinobacteriota, Desulfobacterota, Proteobacteriota, and lactobacilli, as well as species of *Faecalibacterium* and *Bifidobacterium*) can reduce the production of useful metabolites (eg, SCFAs) involved in maintaining intestinal homeostasis and integrity by controlling the defense mechanism. A deficiency of these metabolites can lead to pathological conditions such as colitis, IBD, and even colorectal cancers.^89^^,^^90^ Ruminococcaceae produce butyrate that regulates mucosal immunity and is the best indicator of healthy anaerobic gut microflora. Butyrate limits the production of proinflammatory cytokines like tumor necrosis factor-α, thus reducing the risk of inflammatory bowel disease (IBD).^91^

### Correlation between the metabolic changes and gut microbiota composition in response to Fluoride

The current review found that the metabolic changes in the participants were closely related to changes in microflora composition after fluoride intake. The increased expression of proinflammatory cytokines and antimicrobial peptides followed by a decrease in antioxidative enzymes and tight junction proteins, for example, can be described by a lower abundance of lactobacilli, *Bifidobacterium, Clostridium*, and *Faecalibacterium*, which was responsible for decreased SCFAs levels—specifically, butyrate content that maintains balance in the production of proinflammatory cytokines. These changes resulted in increased expression of proinflammatory cytokines, such as *IL-17A* and *IL-22*,^10^^,^^37^ and mucosal sIgA antibody,^34^^,^^36^ that further led to the production of antimicrobial peptides such as *CRAMP, β-defensin-1*, and *β-defensin-3*, consequently leading to inflammatory diseases like IBD.^10^ The changes also were associated with a reduction in levels of glycoproteins and antioxidant enzymes such as *SOD*, catalase, and *GSH*, which may cause colorectal cancer and is linked with impairment in the integrity of the intestinal barrier.^10^^,^^37^^,^^49^^,^^54^ Additionally, a decrease in the mRNA levels of *BDNF*, cAMP-responsive element, and stem cell factor were observed, which are an important modulators of neuroplasticity, thus leading to neurodevelopmental disorders.^50^  *IL-17A and IL-22* are cytokines often produced together at high levels in inflamed tissues. Cytokines provide a protective inflammatory response, but dysregulation can cause autoimmunity and allergies.^92^ Increased cytokine production modulates the secretion of *β-defensins* that regulate innate immunity, thus inducing inflammatory mediators. *β-Defensins* maintain the balance between safeguarding from pathogens and tolerance to normal flora. Nevertheless, attenuated expression triggers the activation of transcription factor *NF-κB* and, in turn, activates the inflammatory pathways.^93^ Mucosal *sIgA* antibody serves as the first line of defense against pathogens, and its increased production is an indicator of an existing underlying disease.^94^ The antioxidant enzymes such as *SOD* protect the cell from oxidative stress, CAT breakdown, or decompose hydrogen peroxide molecules into water, thereby protecting cells and organs from damage caused by oxygen-free radicals. Glutathione is involved in tissue repair. The reduced expression of these enzymes might be due to the reduced levels of probiotics, especially the lactobacilli and *Bifidobacterium*, which produce SCFAs, that play a key role in reducing oxidative stress by activating the transcription of antioxidative pathways.^95^^,^^96^ Similarly, several studies showed that imbalances in the proportion of certain bacteria, such as *Clostridium sensu stricto*, *Romboutsia*, Lachnospiraceae, *Desulfovibrio*, and 2 unidentified genera in the Muribaculaceae and Ruminococcaceae families, after fluoride exposure increased the circulating levels of bacterial metabolites such as trimethylamine-*N*-oxide, and an upregulation of mRNA expression levels of pro-inflammatory cytokines, *CRAMP, β-defensin-1, β-defensin-3*, and light chain 3, which were strongly correlated with the higher risk of cardiovascular events. *Desulphovibrio* spp are associated with the production of propionic acid and hydrogen sulfide, a probable mitochondrion toxin, resulting in both neurotoxic and colonotoxic effects.^10^ Furthermore, an increase in the abundance of *Parabacteroides* increased the expression levels of Beclin 1, light chain 3, autophagy protein 5, and *p62* that not only induced autophagy but also altered the levels of luteinizing hormone and follicle-stimulating hormone, thus leading to lower testicular organ functioning and nephrotoxicity.^40^^,^^46^

Most of the discussed studies also reported that fluoride may induce obesity and colitis along with IBD. This can be described by the disturbances in the Firmicuteota to Bacteroidota ratio. The abrupt increase or decrease in the abundance of Firmicuteota or Bacteroidota can lead to obesity and colitis.^41^^,^^46^ Fluoride intervention increased the relative abundance of Firmicuteota and decreased the Bacteroidota population, leading to the elevated Firmicuteota to Bacteroidota ratio. This disturbance can promote the growth of other opportunistic bacteria, such as Proteobacteria, and downregulate the abundance of SCFA-producing bacteria such as members of the Ruminococcaceae, a better indicator of healthy gut microbiota^41^^,^^46^ and *Turicibacter*. This disturbance causes the suppression of SCFAs such as butyrate, propionate, and acetate that play an important role in prevention of obesity and colitis by interacting with adipose tissues via G-protein coupled receptors (GPRs) expressed in adipocytes. The upregulation of *GPR41* and *GPR43* promotes adipocyte formation and inhibits lipolysis, thus inhibiting colitis.^97^ Furthermore, *Turicibacter* controls the host bile acid profile and is considered a modulator of host fat biology and of glucose and energy metabolism. The depletion of *Turicibacter* might be associated with obesity and colitis and correlated with decrease in butyrate levels.^41^^,^^46^

### Factors Affecting Exposure Effects of Fluoride

#### Effects of Fluoride Dose

The dose of fluoride through diet or water has a discerning effect on the stimulation or inhibition of gut microbiomes. Three-fourths of the studies included in this systematic review used high doses of fluoride (>50 mg L^–1^ NaF for animals, and 10-15 mg L^–1^ NaF for humans). Nevertheless, 1 human (1-2 mg L^–1^ NaF) and 4 animal (0.5, 4, 5, and 10 mg L^–1^ NaF in water) studies^34^^,^^44^^,^^48^^,^^53^ also used applicable doses and observed an increase in the growth of probiotics like lactobacilli and *Bifidobacterium*. However, when the fluoride supplemental doses were acutely higher, the abundance of pathogenic microorganisms increased, and health-promoting microbial species decreased. Numerous in vitro and in vivo studies also showed dose-dependent effects of fluoride supplementation on gut microbiota compositional changes.^10^^,^^31^^,^^34^^,^^35^^,^^41^^,^^42^^,^^46^^,^^48^^,^^54^^,^^71–73^ Overall, doses of fluoride higher than 25 mg L^–1^ NaF in animals and 2 mg L^–1^ NaF in humans could disturb microbial homeostasis, which could lead to the upregulation of proinflammatory metabolites expression and downregulation of antioxidative enzymes and junction proteins, thus triggering immune responses and intestinal integrity.

Identifying the crossroads of fluoride dose for these microbes is necessary to reveal underlying biological functions beneficial for health. From the current literature, a fluoride dose of < 50 mg L^–1^ for animals and < 10 mg L^–1^ NaF for humans was recommended to stimulate the abundance of health-promoting strains and decrease the abundance of possible pathogenic strains without affecting other crucial microbes. However, determining the turning-point dose value of fluoride precisely is challenging, due to varied exposure from diet and drinking water, in addition to the diversity in gut barrier structure and fluoride metabolism of the animals and humans in the included studies.

#### Effects of Fluoride’s Duration of Exposure

Greater than 70% of the animal studies focused on long-term exposure to high concentrations of fluoride; however, some in vitro studies (8%) examined the acute effects of fluoride on the microbiome after short-term exposure (24-36 hours).^35^^,^^68^^,^^69^^,^^71^ Although the impacts of long-term exposure and low concentrations are not understood, exposure to doses > 25 mg L^–1^ NaF in water for longer than 1 month in animals, and > 2 mg L^–1^ NaF for > 1 day in humans can lead to severe health conditions. However, the results are not uniform among the studies, due to the diversity of animal species, different biomarkers used for measuring the outcomes, and varying quality of studies.

### Limitations and Knowledge Gaps

This systematic review disentangles the effects of fluoride on gut microbiota and its associated functions with good reliability, due to high-quality assessment scores for most studies and specificity due to the focus on gut microbiota within the inclusion criteria.

However, there are a limited number of human studies exploring the effects of fluoride on the gut microbiome, making it difficult to draw conclusions. Most of the included studies were animal based and used very high doses of fluoride (25 mg L^–1^ NaF in water) compared with the safe intake recommended by WHO (0.8–1.7 mg L^–1^ NaF in water) in humans. Though animals are a useful tool for assessing the impacts of fluoride, extrapolating findings to humans can be difficult due to differences in fluoride sensitivity, intestinal barrier functions, and metabolic rates. Additionally, including all eligible studies led to high inconsistency among the reported outcomes, thus preventing pooling of results.

The mechanisms linking changes in bacterial composition to metabolic dysfunction have not been confirmed, meaning the exact process by which fluoride intervention modulates the gut microbiota and alters SCFAs remains unknown. To further investigate, individual in vitro analyses that includes minimum inhibitory concentrations, minimum bactericidal concentrations, and time-kill curves of strains found in the gut under different fluoride concentrations are needed. The strains under different treatments can be subjected to RNA sequencing and mass spectrometry to identify which genes and proteins are expressed in association with fluoride exposure. This would also help identify the individual response of strains and to obtain a turning-point dose and duration of fluoride exposure. Furthermore, considering the sensitivity of the research area, careful examination of fluoride’s impact in water and diet at low intakes for a short term and long term in human models must be conducted. To better assess the connection, follow-up studies should be considered to investigate whether fluoride-linked microbiome change directly affects the expression of metabolites and human health.

## CONCLUSIONS

Current analysis shows that fluoride intervention either in vivo or in vitro may change the abundance of gut microbiota and its associated activities. Fluoride at low levels (<2 mg L^–1^ NaF in humans and <25 mg L^–1^ NaF in animals) did not affect gut microbiota. Although effects at high doses are inconsistent, impacts could be seen in overall microbial diversity change, change in relative abundance of specific taxa, and changes in the metabolism of present microbiota. Shifts in any of these aspects of the microbial community can lead to health implications. Therefore, more fundamental studies are needed to fully understand the impacts of fluoride at low doses, but for a long duration, as might be expected to be found in diet and water supplies, on key gut microbes such as the lactobacilli and *Bifidobacterium*. More studies are needed to determine the ideal concentration of fluoride for supplementation, in addition to assessing the durability of this effect. These recent findings should stimulate discussions on the safe use of fluoride in food, water, and dietary supplements.

## Supplementary Material

nuae202_Supplementary_Data
