# Supplementary material for: Effect of Fluoride on Gut Microbiota: A Systematic Review
Source: Nutr Rev. 2025 Mar 10;83(7):e1853–80. doi: 10.1093/nutrit/nuae202 (PMC12166178; doi:10.1093/nutrit/nuae202)
Supplement: nuae202_Supplementary_Data [file nuae202_supplementary_data.zip › Supplementary figure 1-Quality Assessment (MMAT).pdf]

| First Author and Reference | Clear Research Questions | Data address research questions | 3.1. Is randomization appropriately performed? | 3.2. Are the groups comparable at baseline? | 3.3. Are there complete outcome data? | 3.4. Are outcome assessors blinded to the intervention provided? | 3.5. Did the participants adhere to the assigned intervention? | 4.1. Is the sampling strategy relevant to address the research question? | 4.2. Is the sample representative of the target population? | 4.3. Are the measurements appropriate? | 4.4. Is the risk of nonresponse bias low? | 4.5. Is the statistical analysis appropriate to answer the research question? | 4.6. Do the different components of the study adhere to the quality criteria of each tradition of the methods involved? | 5.1. Are the participants representative of the target population? | 5.2. Are measurements appropriate regarding both the outcome and intervention (or exposure)? | 5.3. Are there complete outcome data? | 5.4. Are the confounders accounted for in the design and analysis? | 5.5. During the study period, is the intervention administered (or exposure occurred) as intended? | 6.1. Is the sampling strategy relevant to address the research question? | 6.2. Is the sample representative of the target population? | 6.3. Are the measurements appropriate? | 6.4. Is the risk of nonresponse bias low? | 6.5. Is the statistical analysis appropriate to answer the research question? | 6.6. Do the different components of the study adhere to the quality criteria of each tradition of the methods involved? | 8.1. Is the sampling strategy relevant to address the research question? | 8.2. Is the sample representative of the target population? | 8.3. Are the measurements appropriate? | 8.4. Is the risk of nonresponse bias low? | 8.5. Is the statistical analysis appropriate to answer the research question? | 8.6. Do the different components of the study adhere to the quality criteria of each tradition of the methods involved? |
|---|---|---|---|---|---|---|---|---|---|---|---|---|---|---|---|---|---|---|---|---|---|---|---|---|---|---|---|---|---|---|
| **Randomized Controlled trials** | | | | | | | | | | | | | | | | | | | | | | | | | | | | | | |
| Cao, 2019 | Yes | Yes | Yes | Yes | Yes | Yes | Yes | | | | | | | | | | | | | | | | | | | | | | | |
| Dionizio, 2021 | Yes | Yes | Yes | Yes | Yes | Yes | Yes | | | | | | | | | | | | | | | | | | | | | | | |
| Dutta, 2018 | Yes | Yes | Yes | Yes | Yes | Yes | Yes | | | | | | | | | | | | | | | | | | | | | | | |
| Fu, 2020 | Yes | Yes | Yes | Yes | Yes | Yes | Yes | | | | | | | | | | | | | | | | | | | | | | | |
| Fu, 2022 | Yes | Yes | Yes | Yes | Yes | Yes | Yes | | | | | | | | | | | | | | | | | | | | | | | |
| Komuroglu, 2021 | Yes | Yes | Yes | Yes | Yes | Yes | Yes | | | | | | | | | | | | | | | | | | | | | | | |
| Li 2021 | Yes | Yes | Yes | Yes | Yes | Yes | Yes | | | | | | | | | | | | | | | | | | | | | | | |
| Li 2016 | Yes | Yes | Yes | Yes | Yes | Yes | Yes | | | | | | | | | | | | | | | | | | | | | | | |
| Liu 2019 | Yes | Yes | Yes | Yes | Yes | Yes | Yes | | | | | | | | | | | | | | | | | | | | | | | |
| Liu 2021 | Yes | Yes | Yes | Yes | Yes | Yes | Yes | | | | | | | | | | | | | | | | | | | | | | | |
| Luo 2016 | Yes | Yes | Yes | Yes | Yes | Yes | Yes | | | | | | | | | | | | | | | | | | | | | | | |
| Miao 2020 | Yes | Yes | Yes | Yes | Yes | Yes | Yes | | | | | | | | | | | | | | | | | | | | | | | |
| Miao 2020 | Yes | Yes | Yes | Yes | Yes | Yes | Yes | | | | | | | | | | | | | | | | | | | | | | | |
| Pimentel 2019 | Yes | Yes | Yes | Unclear | Unclear | Yes | Yes | | | | | | | | | | | | | | | | | | | | | | | |
| Qiu 2020 | Yes | Yes | Yes | Yes | Yes | Yes | Yes | | | | | | | | | | | | | | | | | | | | | | | |
| Sun 2020 | Yes | Yes | Yes | Yes | Yes | Yes | Yes | | | | | | | | | | | | | | | | | | | | | | | |
| Wang 2019 | Yes | Yes | Yes | Yes | Yes | Yes | Yes | | | | | | | | | | | | | | | | | | | | | | | |
| Wang 2020 | Yes | Yes | Yes | Yes | Yes | Yes | Yes | | | | | | | | | | | | | | | | | | | | | | | |
| Xin 2021 | Yes | Yes | Yes | Yes | Yes | Yes | Yes | | | | | | | | | | | | | | | | | | | | | | | |
| Xin 2021 | Yes | Yes | Yes | Yes | Yes | Yes | Yes | | | | | | | | | | | | | | | | | | | | | | | |
| Yan 2021 | Yes | Yes | Yes | Yes | Unclear | Yes | Yes | | | | | | | | | | | | | | | | | | | | | | | |
| Yasuda 2017 | Yes | Yes | Yes | Yes | Unclear | Yes | Unclear | | | | | | | | | | | | | | | | | | | | | | | |
| Yu 2021 | Yes | Yes | Yes | Yes | Yes | Yes | Yes | | | | | | | | | | | | | | | | | | | | | | | |
| Zhang 2022 | Yes | Yes | Yes | Yes | Unclear | Yes | Yes | | | | | | | | | | | | | | | | | | | | | | | |
| Zhong 2022 | Yes | Yes | Yes | Yes | Yes | Yes | Yes | | | | | | | | | | | | | | | | | | | | | | | |
| Zhu 2022 | Yes | Yes | Yes | Yes | Yes | Yes | Yes | | | | | | | | | | | | | | | | | | | | | | | |
| Zhang 2023 | Yes | Yes | Yes | Yes | Yes | Yes | Yes | | | | | | | | | | | | | | | | | | | | | | | |
| Tian 2023 | Yes | Yes | Unclear | Unclear | No | Yes | Yes | | | | | | | | | | | | | | | | | | | | | | | |
| Haonan Huang, 2023 | Yes | Yes | Yes | Yes | Yes | Yes | Yes | | | | | | | | | | | | | | | | | | | | | | | |
| Wu, Yue, 2024 | Yes | Yes | Yes | Yes | Yes | Yes | Yes | | | | | | | | | | | | | | | | | | | | | | | |
| Chenjun Zhao, 2024 | Yes | Yes | Yes | Yes | Yes | Yes | Yes | | | | | | | | | | | | | | | | | | | | | | | |
| ZHANG Xiao Li, 2023 | Yes | Yes | Yes | Yes | Yes | Yes | Yes | | | | | | | | | | | | | | | | | | | | | | | |
| Dashuan, 2023 | Yes | Yes | Yes | Yes | Yes | Yes | Yes | | | | | | | | | | | | | | | | | | | | | | | |
| Guijie Chen, 2023 | Yes | Yes | Yes | Yes | Yes | Yes | Yes | | | | | | | | | | | | | | | | | | | | | | | |
| Taotao Zhao, 2024 | Yes | Yes | Yes | Yes | Yes | Yes | Yes | | | | | | | | | | | | | | | | | | | | | | | |
| Zhe Mo,2023 | Yes | Yes | Yes | Yes | Yes | Yes | Yes | | | | | | | | | | | | | | | | | | | | | | | |
| Licai Shi, 2020 | Yes | Yes | Yes | Yes | Yes | Yes | Yes | | | | | | | | | | | | | | | | | | | | | | | |
| Yueying Feng, 2024 | Yes | Yes | Yes | Yes | Yes | Yes | Yes | | | | | | | | | | | | | | | | | | | | | | | |
| **Experimental/Lab based studies** | | | | | | | | | | | | | | | | | | | | | | | | | | | | | | |
| Chen, 2021 | Yes | Yes | | | | | | Yes | Yes | Yes | Yes | Yes | Yes | | | | | | | | | | | | | | | | | |
| Davis,2012 | Yes | Yes | | | | | | Yes | Yes | Unclear | Yes | Unclear | Unclear | | | | | | | | | | | | | | | | | |
| Li, 2020 | Yes | Yes | | | | | | Yes | Yes | Yes | Yes | Yes | Yes | | | | | | | | | | | | | | | | | |
| Ma 2014 | Yes | Yes | | | | | | Yes | Yes | Yes | Yes | Yes | Yes | | | | | | | | | | | | | | | | | |
| Parthasaradhi, 2018 | Yes | Yes | | | | | | Yes | Yes | Yes | Yes | Unclear | Yes | | | | | | | | | | | | | | | | | |
| Parthasaradhi, 2020 | Yes | Yes | | | | | | Yes | Unclear | Yes | Yes | Yes | Unclear | | | | | | | | | | | | | | | | | |
| **Non radomised control trials** | | | | | | | | | | | | | | | | | | | | | | | | | | | | | | |
| Li, 2022 | Yes | Yes | | | | | | | | | | | | Yes | Yes | Yes | Unclear | Yes | | | | | | | | | | | | |
| **Cohort Studies** | | | | | | | | | | | | | | | | | | | | | | | | | | | | | | |
| Hai-Jeon Yoon, 2019 | Yes | Yes | | | | | | | | | | | | | | | | | Yes | Yes | Yes | Yes | Yes | Yes | | | | | | |
| **Case- control Study** | | | | | | | | | | | | | | | | | | | | | | | | | | | | | | |
| Zhou, 2023 | Yes | Yes | | | | | | | | | | | | | | | | | | | | | | | Yes | Yes | Yes | Yes | Yes | Yes |
| Wang 2023 | | | | | | | | | | | | | | | | | | | | | | | | | Yes | Yes | Unclear | Yes | Unclear | Unclear |

Legend: Yes (green), No (red), Unclear (yellow)

**Figure 3 :** Quality assessment scores for included articles using the Mixed Methods Assessment Tool
