# Supplementary material for: Effect of Fluoride on Gut Microbiota: A Systematic Review
Source: Nutr Rev. 2025 Mar 10;83(7):e1853–80. doi: 10.1093/nutrit/nuae202 (PMC12166178; doi:10.1093/nutrit/nuae202)
Supplement: nuae202_Supplementary_Data [file nuae202_supplementary_data.zip › supplementary tables updated.docx]

**Supplementary Table 1 (S1): Search Strategy**

| **Data Source** | **Search strategy** | | | | | **Number of hits** |
| --- | --- | --- | --- | --- | --- | --- |
| **Web of Science** | **Terms connected by ‘OR’** | **AND** | **Terms connected by ‘OR’** | **AND** | **Terms connected by ‘OR’** | 478 |
|  | Gut  **OR** gastrointestinal **OR** "gastro intestinal" **OR** intestin* **OR** colon*** OR** bowel***** |  | microbiota **OR** "micro biota" **OR** microbiome **OR** "micro biome" **OR** flora **OR** microflora **OR** "micro flora" **OR** bacteria |  | fluorid* **OR**  fluori* |  |
| **PubMed** | "gastrointestinal microbiome"[MeSH Terms] **OR** "gastrointestinal" |  | "microbiome" **OR** "gastrointestinal microbiome" **OR** "gut" **OR** "microbiota” **OR** "gut microbiota" |  | "fluoridate" **OR** "fluoridated" **OR** "fluoridating" **OR** "fluoridation"[MeSH Terms] **OR** "fluoridation" **OR** "fluoridation's" **OR** "fluoride's" **OR** "fluorided" **OR** "fluorides"[MeSH Terms] **OR** "fluorides" **OR** "fluoride" **OR** "fluoridization" **OR** "fluoridized" | 42 |
| **Scopus** | Gut **OR** gastrointestinal **OR** " gastrointestinal" **OR** intestin***OR** colon* **OR** bowel* | **W/2** | Microbiota **OR** "microbiota" **OR** microbiome **OR** "microbiome" **OR** flora OR microflora **OR** "microflora" **OR** bacteria |  | fluorid* **OR**  fluori* | 260 |

**Supplementary Table 2 (S2) : Search strategy**

| **Data Source** | **Search strategy** | | **Number of hits** |
| --- | --- | --- | --- |
| **Embase<1974to2022June 24>** | **1** | Exp intestine flora/ | **115199** |
|  | **2** | ((gut or gastrointestinal or gastro intestinal or intestin*or colon*or bowel*) adj2 (microbiota or micro biota or microbiome or micro biome or flora or microflora or micro flora or bacteria)).ti,ab,kw. | **86025** |
|  | **3** | 1or2 | **136989** |
|  | **4** | fluoride/ | **34473** |
|  | **5** | fluoridation/ | **4640** |
|  | **6** | fluorid*.ti,ab,kw. | **60698** |
|  | **7** | or/4-6 | **69630** |
|  | **8** | 3and7 | **57** |
|  | **9** | fluori* ti,ab,kw. | **96402** |
|  | **10** | **3 and 9** | **102** |
| **Medline** | **S1** | (MH"GastrointestinalMicrobiome") | **31,704** |
|  | **S2** | (gut or gastrointestinal or "gastro-intestinal" or intestin* or colon*or bowel*) N2(microbiota or "microbiota" or microbiome or "microbiome" or flora or microflora or "microflora" or bacteria) | **81,011** |
|  | **S3** | S1 ORS 2 | **81,011** |
|  | **S4** | (MH"Fluorides+") | **38,843** |
|  | **S5** | (MH"Fluoridation") | **5,913** |
|  | **S6** | fluorid* | **68,014** |
|  | **S7** | S4 OR S5 OR S6 | **70,525** |
|  | **S8** | **S3 AND S7** | **100** |
| **CINAHL** | **S1** | ("Gastrointestinal Microbiome" or microbiota) | **12,184** |
|  | **S2** | (gut or gastrointestinal or "gastro-intestinal" or intestin* or colon* or bowel*) N2 (microbiota or "micro biota" or microbiome or "micro biome" or flora or microflora or "micro flora" or bacteria) | **11,489** |
|  | **S3** | S1 OR S2 | **15,146** |
|  | **S4** | ("Fluorides") | **4,442** |
|  | **S5** | ("Fluoridation") | **1,606** |
|  | **S6** | fluorid* or fluori* | **7,647** |
|  | **S7** | S4 OR S5 OR S6 | **7,647** |
|  | **S8** | **S3 AND S7** | **22** |

**Supplementary Table 3 (S3) : Summary of Included Studies**

| **Study ID** | **Country of Origin** | **Study Design** | **Quality assessment criteria met** | **Sample size** | **Population** | **Assessment time points** | **Duration of Exposure** | **Biomarkers** |
| --- | --- | --- | --- | --- | --- | --- | --- | --- |
| Cao, 2019 | China | Randomised control design | **5** | n= 18 (control arm n= 9, treatment arm n= 9) | Mice | 60 days | 60 days | Faeces |
| Davis, 2012 | Australia | Experimental study/ Invitro | **3** | N/A | Bovine | Baseline and after 24, 48 and 72 hours | 72 hrs | Cattle rumen |
| Dionizio, 2021 | Australia | Randomised control design | **5** | n= 18 (control arm n= 6, treatment arm n= 6+6) | Rats (*Rattus norvegicus*, Wistar) | 30days | 30days | Ileum |
| Dutta, 2018 | India | Randomised control design | **5** | N/A | *Drosophila Melanogaster* | 24 hrs | 24 hrs | Mid Gut |
| Fu, 2020 | China | Randomised control design | **5** | n= 18 (control arm n= 9, treatment arm n= 9) | ICR mice | 60 days | 60 days | Faeces |
| Fu, 2022 | China | Randomised control design | **5** | n=48 (control arm n= 12, treatment arm n= 36)  100 mg/ NaF n= 12  Exercise n=12  Exercise+ NaF n=12 | ICR mice | 6 months | 6 months | Duodenum and Colon |
| Komuroglu, 2021 | Turkey | Randomised control design | **5** | n= 14 (control arm n= 7, treatment arm n= 7) | Male Wistar albino rats | 12 weeks | 12 weeks | Intestinal tissue |
| Li, 2021 | China | Randomised control design | **5** | n= 14 (control arm n= 7, treatment arm n= 7) | Ducklings | 28 days | 28 days | Intestinal tissue |
| Li, 2020 | China | Experimental study/ Invitro | **6** | n= 6 (control arm n= 3, treatment arm n= 3) | Silkworm | As the study is an in vitro study , it has taken place at different time points and duration depending upon the experimental tests to be performed | As the study is an in vitro study , it has taken place at different time points and duration depending upon the  tests to be performed | Intestinal Content |
| Li, 2016 | China | Randomised control design | **5** | n= 12 (control arm n= 6, treatment arm n= 6) | Silkworm larvae (734 strain and T6 strain) | Every 10 mins from the fourth  instar to the third day of fifth instar. | Every 10 mins from the fourth  instar to the third day of fifth instar. | Intestinal Content |
| Li, 2022 | China | Non-Randomised control design | **4** | n= 6 (control arm n= 3, treatment arm n= 3) | Silkworm larvae (Bombyx mori) | 36 hours | 36 hours | Intestinal Content |
| Liu, 2019 | China | Randomised control design | **5** | n= 72 (control arm n= 24, treatment arm n= 48)  110.5 mg NaF, =50 mg F ion/L, n= 24  221 mg NaF,=100 mg F ion/L , n= 24 | Kunming Mice | 70 days | 70 days | Cecal Contents |
| Liu, 2021 | China | Randomised control design | **5** | n= 24 (control arm n= 6, treatment arm n= 18)  100 mg/l NaF, n= 6  50 mg/L NaAsO2, n= 6  100 mg/L NaF +50 mg/L NaAsO2, n=6 | Sprague-Dawley rats | 69 days | 69 days | Faeces |
| Luo, 2016 | China | Randomised control design | **5** | n= 280 (control arm n= 70, treatment arm n= 210)  400 mg F/kg, n= 70  800 mg F/kg, n= 70  1200 mg F/kg, n=70 | Broiler chicken | After 21 and 42 days | 42 days | Ileum and cecal digesta |
| Ma, 2014 | China | Experimental study/ Invitro | **6** | n= 15 (control arm n= 3, treatment arm n= 12)  Three for each of the four treatments | N/A | After 6 hours | 6 hours | N/A |
| Miao, 2020 (a) | China | Randomised control design | **5** | n= 288 (control arm n= 96, treatment arm n= 192)  400 (low-F) mg/kg F, n= 96  1200 mg/kg F (high-F), n= 96 | Laying Hens | 16 hours | 16 hours | Small intestine and cecal contents |
| Miao, 2020 (b) | China | Randomised control design | **5** | n= 288 (control arm n= 96, treatment arm n= 192)  400 (low-F) mg/kg F, n= 96  1200 mg/kg F (high-F), n= 96 | Laying Hens | 59 days | 59 days | Small intestine and cecal contents |
| Parthasaradhi, 2018 (a) | India | Experimental study/ Invitro | **5** | n=12 (control arm n=2 treatment arm n= 10) | N/A | 24 hrs for MIC and 30 hours for growth curves | 24 hrs for MIC and 30 hours for growth curves | N/A |
| Parthasaradhi, 2018 (b) | India | Experimental study/ Invitro | **4** | n=8  (Control arm n=4 treatment arm n= 4) | N/A | 24 hrs | 24 hrs | N/A |
| Pimentel, 2019 | Brazil | Randomised control design | **3** | n=24  (Control arm n=6 treatment arm n= 18)  6 for each of the three groups | Bovine and mice | 7 days | 7 days | Rumen fluid |
| Qiu, 2020 | United States | Randomised control design | **5** | n=18  (Control arm n=3  Treatment arm  50 mg/L NaAsO2 n=3  100 mg/L NaF n= 3  50 mg/L NaAsO2and100 mg/L NaF n=3 | Offspring rats | 121 days | 121 days (as at first the parents were given fluoride for 21 days and then springs were treated with fluoride until postnatal day 90.) | Faeces |
| Sun, 2020 | China | Randomised control design | **5** | n=24  (Control arm n=8  Treatment arm n=16  100 mg/L NaF n=8  100 mg/L NaF+ Probiotic n=8 | ICR male mice | 70 days | 70 days | Faeces |
| Wang, 2020 | China | Randomised control design | **5** | n=72  (Control arm n=24  Treatment arm n=48  100 mg/L F n=24  50 mg/L F n=24 | Kunming mice | 70 days | 70 days | Rectal contents |
| Wang, 2019 | China | Randomised control design | **5** | n= 120  (Control arm n= 30  Treatment arm n=90  0.5 mg/L NaF n=30  5 mg/L NaF n=30  50 mg/L NaF n=30 | *Bufo gargarizans* tadpoles | The outcomes were assessed after 105 days from gosner stage 26 to 38 | 105 days | gut |
| Xin, 2021 | China | Randomised control design | **5** | n=30  (Control arm n=10  Treatment arm n=20  100 mg/L NaF n=10  100 mg/L NaF+ Probiotic n=10 | Mice | 98 days | 98 days | Kidney and colon |
| Xin, 2021 | China | Randomised control design | **5** | n= 108  (Control arm n=36  Treatment arm n=72  100 mg/L NaF 100 ppm NaF ≈ 37.8 ±2.4 ppm F ̅ n=36  L. johnsonii BS15 (prob group; 0.2 mL/day) n=36 | Mice | 70 days | 70 days | Ileal content |
| Yan, 2021 | China | Randomised control design | **4** | n= 108  (Control arm n=36  Treatment arm n=72  100 mg/L NaF 100 ppm NaF ≈ 37.8 ±2.4 ppm F ̅ n=36  *L. johnsonii* BS15 (prob group; 0.2 mL/day) n=36 | Offspring Rats | 121 days | 121 days | Faeces |
| Yasuda, 2017 | USA | Randomised control design | **3** | n= 17  6 for oral and 11 for stool  (Control arm n=6  3 for oral and 3 for stool  Treatment arm n=12  3 for high fluorine group oral and 2 and 3 samples each for high and low fluoride group. | wild-type BALB/c mice | Outcomes were assessed at 0, 4, 8, and 12 weeks. | 12 weeks | Faeces and Saliva |
| Yu, 2020 | China | Randomised control design | **5** | n= 80  (Control arm n=40  Treatment arm n= 40) | Common carp | 90 days | 90 days | Intestinal tissue |
| Zhang, 2022 | China | Randomised control design | **4** | n= 900  (Control arm n=180  Treatment arm n= 720)  180 for each of 4 treatments | Zebrafish | Assessed after 30,60 and 90 days | 90 days | Faeces |
| Zhong, 2022 | China | Randomised control design | **5** | n= 25  (Control arm n=5  Treatment arm n= 20)  5 for each of 4 treatments | Rats | 12 weeks | 12 weeks | Faeces |
| Zhu, 2022 | China | Randomised control design | **5** | n= 72  (Control arm n=18  Treatment arm n= 54)  18 for each of 3 treatments | Kunming mice | 70 days | 70 days | Colonic content |
| Zhang, 2023 | China | Randomised control design | **5** | n= 108  (Control arm n=12  Treatment arm n= 96)  12 for each of 8 treatments | ICR mice | 90 days | 90 days | Faeces |
| Zhou, 2023 | China | Case-control study | **6** | n= 19  (Control arm n= 10, treatment arm n=9) | Sprague-Dawley (SD) rats | 90 days | 90 days | Faeces |
| Tian 2023 | China | Randomised control design | **2** | N/A | Sprague-Dawley (SD) rats | 120 days | 120 days | Faeces |
| Haonan Huang, 2023 | China | Randomised control design | **5** | n=88  (Control arm n= 44, treatment arm n= 44) | Inbred male C57BL/6 J mice | 8 weeks | 8 weeks | colon |
| Wu, Yue, 2024 | China | Randomised control design | **5** | n= 36  (Control arm n= 12, treatment arm n= 24) | C57BL/6J mice | 18 weeks | 18 weeks | Ileum |
| Chenjun Zhao, 2024 | China | Randomised control design | **5** | n= 36  (Control arm n= 12, treatment arm n= 24) | Sprague–Dawley Rats | 6 weeks | 6 weeks | Faeces |
| ZHANG Xiao Li, 2023 | China | Randomised control design | **5** | n= 60; 40 F and 20 M  (Control arm n=, treatment arm n= 24) | Sprague–Dawley Rats | 121 days | 121 days | colon |
| Dashuan, 2023 | China | Randomised control design | **5** | n= 30  (Control arm n= 6, treatment arm n= 6+6+6=24) | Sprague–Dawley Rats | 3 months | 3 months | colon |
| Guijie Chen, 2023 | China | Randomised control design | **5** | n= 16  (Control arm n= 8, treatment arm n= 8) | C57BL/6J mice | 10 weeks | 10 weeks | colon |
| Taotao Zhao, 2024 | China | Randomised control design | **5** | n= 48  (Control arm n= 12, treatment arm n= 12+12+12=36) | ICR mice | 60 days | 60 days | colon |
| Zhe Mo,2023 | China | Randomised control design | **5** | n= 25  (Control arm n= 5, treatment arm n= 5+5+5+5=20) | Wistar rats | 12 weeks | 12 weeks | Faeces |
| Licai Shi, 2020 | China | Randomised control design | **5** | n= 50  (Control arm n= 10, treatment arm n= 10+10+10+10+ 10=40) | C57BL/6J mice | 5 weeks | 5 weeks | Faeces |
| Yueying Feng, 2024 | China | Randomised control design | **5** | n= 30  (Control arm n= 10, treatment arm n= 10+10=20) | C57BL/6J mice | Postnatal 21 days | Postnatal 21 days | Faeces |
|  |  |  |  | **Humans Study** |  |  |  |  |
| **Study ID** | **Country of Origin** | **Study Design** | **Quality assessment criteria met** | **Sample size** | **Population** | **Assessment time points** | **Duration of Exposure** | **Biomarkers** |
| Chen, 2021 | China | Experimental study/ Invitro | **6** | n=15 (control arm n= 3, treatment arm n=12)  Three for each of the 4 treatments | Healthy Human | 24 Hrs | 24 Hrs | Faeces |
| Zhou, 2023 | China | Case-control study | **6** | n=23  (Control arm n= 14, treatment arm n=9) | Children with dental fluorosis | N/A | N/A | Faeces |
| [Hai-Jeon Yoon](javascript:;), 2019 | Korea | Cohort study | **6** | n= 114  (1^ST^ Cohort n=75, 2^nd^n= 39) | Females with breast cancer | N/A | N/A | Faeces |
| Wang, 2023 | China | Case-control study | **3** | n=65  (Control arm n= 33, treatment arm n=32) | Patients with dental fluorosis | N/A | N/A | Faeces |

**Supplementary Table 4 (S4): Intervention doses used *in invitro* and *in vivo***

| **Paper ID** | **In vitro** | **In vivo** | **Dose used** | **Duration** |
| --- | --- | --- | --- | --- |
| **­­­**  **1.**  Cao, 2019  China |  | ✓ | 100 mg/l NaF | 60 days |
| **2.**  Davis,2012  Australia | ✓ |  | 20 mM sodium fluoroacetate (added in media) | 72 hrs |
| **3.**  Dionizio, 2021  Australia |  | ✓ | 10 mg/l F  50 mg/l F | 30 days |
| **4.**  Dutta, 2018  India |  | ✓ | 100 NaF (52µg/mL F)  200 NaF (89.9µg/mL F)  300 NaF (126µg/mL F)  400 NaF (157µg/mL F)  500 NaF (175µg/mL F) | 24 hrs |
| **5.**  Fu, 2020  China |  | ✓ | 100 mg/l NaF | 60 days |
| **6.**  Fu, 2022  China |  | ✓ | 100 mg/l NaF | 6 months |
| **7.**  Komuroglu, 2021  Turkey |  | ✓ | 100 mg/l NaF | 12 weeks |
| **8.**  Li,2021  China |  | ✓ | 750 mg/kg NaF ~ 750 mg/l NaF | 28 days |
| **9.**  Li, 2020  China | ✓ |  | 4.76 mM NaF | As the study is an in vitro study, it has taken place at different time points and durations depending on the  tests to be performed |
| **10.**  Li, 2016  China |  | ✓ | 200mg/kg NaF~ 200 mg/l NaF | Every 10 mins from the fourth  instar to the third day of the fifth instar. |
| **11.**  Li,2022  China |  | ✓ | 200mg/kg NaF~ 200 mg/l NaF | 36 hours |
| **12.**  Liu, 2019  China |  | ✓ | 110.5 mg NaF, =50 mg F ion/L  221 mg NaF, =100 mg F ion/L | 70 days |
| **13.**  Liu,2021  China |  | ✓ | 100 mg/l NaF | 69 days |
| **14.**  Luo, 2016  China |  | ✓ | 400 mg F/kg~ 400 mg/l F  800 mg F/kg~ 800 mg/l F  1200 mg F/kg~ 1200 mg/l F | 42 days |
| **15.**  Ma, 2014  China | ✓ |  | 0.1mM NaF  1 mM NaF  10 mM NaF  100 mM NaF  Supplemented in media | 6 hours |
| **16.**  Miao,2020 (a)  China |  | ✓ | 400 (low-F) mg/kg F~ 400 mg/l F  1200 mg/kg F (high-F) ~ 1200 mg/l F | 16 hours |
| **17.**  Miao,2020 (b)  China |  | ✓ | 400 (low-F) mg/kg F~ 400 mg/l F  1200 mg/kg F (high-F) ~ 1200 mg/l F | 59 days |
| **18.**  Parthasaradhi, 2018 (a)  India | ✓ |  | 10.0 mMNaF  20.0 mM NaF  30.0 mM NaF  40.0 mMNaF  50.0 mMNaF | 24 hrs for MIC and 30 hours for growth curves |
| **19.**  Parthasaradhi, 2020 (b)  India | ✓ |  | 10.0 mMNaF  20.0 mM NaF  30.0 mM NaF  40.0 mMNaF  50.0 mMNaF | 24 hrs |
| **20.**  Pimentel,2019  Brazil |  | ✓ | 0.266mg/kg sodium fluoroacetate (SF)~ 0.266 mg/l SF | 7 days |
| **21.**  Qiu,2020  United States |  | ✓ | 100 mg/l NaF | 121 days (as at first the parents were given fluoride for 21 days and then springs were treated with fluoride until postnatal day 90.) |
| **22.**  Sun,2020  China |  | ✓ | 100 mg/l NaF | 70 days |
| **23.**  Wang, 2020  China |  | ✓ | 100 mg/l NaF and 50 mg/l NaF | 70 days |
| **24.**  Wang, 2019  China |  | ✓ | 0.5 mg/L NaF  5 mg/L NaF  50 mg/L NaF | 105 days |
| **25.**  Xin,2021  China |  | ✓ | 100 mg/l NaF | 98 days |
| **26.**  Xin,2021  China |  | ✓ | 100 ppm (mg/l) NaF ≈ 37.8 ±2.4 ppm F ̅ | 70 days |
| **27.**  Yan,2021  China |  | ✓ | 100 mg/l NaF | 121 days |
| **28.**  Yasuda, 2017  USA |  | ✓ | 4ppm (mg/l) F in drinking water | 12 weeks |
| **29.**  Yu, 2021  China |  | ✓ | 80 mg/l NaF | 90 days |
| **30.**  Zhang, 2022  China |  | ✓ | 80 mg/l NaF | 90 days |
| **31.**  Zhong, 2022  China |  | ✓ | 25 mg/L (NaF)  50 mg/L (NaF)  100 mg/L (NaF)  150 mg/L (NaF) | 12 weeks |
| **32.**  Zhu, 2022  China |  | ✓ | 25 mg/L (NaF)  50 mg/L (NaF)  100 mg/L (NaF) | 70 days |
| **33.**  Zhang, 2023  China |  | ✓ | 100 mg/l NaF | 90 days |
| **34.**  Zhou, 2023  China |  | ✓ | 100 mg/l NaF | 90 days |
| **35.**  Tian, 2023  China |  | ✓ | 100 mg/l NaF | 120 days |
| **36.**  Haonan Huang, 2023  China |  | ✓ | 24 mg/kg NaF ≡ 24 mg/L NaF | 8 weeks |
| **37.**  Wu, Yue, 2024  China |  | ✓ | 100 mg/l NaF | 18 weeks |
| **38.**  Chenjun Zhao, 2024  China |  | ✓ | 200 mg/l NaF | 6 weeks |
| **39.**  ZHANG Xiao Li, 2023  China |  | ✓ | 100 mg/l NaF | 121 days |
| **40.**  Dashuan, 2023  China |  | ✓ | 15 mg/kg F (15 mg/l F)  45 mg/kg F (45 mg/l F)  75 mg/kg F (75 mg/l F) | 3 months |
| **41.**  Guijie Chen, 2023  China |  | ✓ | 50 mg/l NaF | 10 weeks |
| **42.**  Taotao Zhao, 2024  China |  | ✓ | 100 mg/l NaF | 60 days |
| **43.**  Zhe Mo,2023  China |  | ✓ | 25 mg/L (NaF)  50 mg/L (NaF)  100 mg/L (NaF)  150 mg/L (NaF) | 12 weeks |
| **44.**  Licai Shi, 2020  China |  | ✓ | 0.5 mg/kg (mg/l) Perfluorooctanoic Acid  1 mg/kg (mg/l) Perfluorooctanoic Acid  3 mg/kg (mg/l) Perfluorooctanoic Acid | 5 weeks |
| **45.**  Yueying Feng, 2024  China |  | ✓ | 0.57 mg/L F35  5.7 mg/L F35 | Postnatal 21 days |
|  |  | **Human Studies** |  |  |
| **Paper ID** | **In vitro** | **In vivo** | **Dose used** | **Duration** |
| **1.**  Chen, 2021  China | ✓ |  | 1 mg/l F  2 mg/l F  10 mg/l F  15 mg/l F | 24 Hrs |
| **2.**  Zhou, 2023  China |  | ✓ | no dose mentioned | N/A |
| **3.**  [Hai-Jeon Yoon](javascript:;), 2019  Korea |  | ✓ | Fluorine-18-fluorodeoxyglucose (18F-FDG) intestinal uptake no dose mentioned | N/A |
| **4.**  Wang, 2023  China |  | ✓ | no dose mentioned | N/A |

**Supplementary Table 5 (S5): Summary of studies showing an effect of fluoride on alpha diversity**

| **Study ID** | **Dose of Fluoride** | **Assessment time points** | **Duration of Exposure** | **Biomarkers** | **Methods used for assessing microbial richness and results** | | | | **Microbial alpha diversity method and results** | | | |
| --- | --- | --- | --- | --- | --- | --- | --- | --- | --- | --- | --- | --- |
|  |  |  |  |  | **OTUs** | | **Chao 1 index** | | **Simpson diversity** | | **Shannon diversity** | |
|  |  |  |  |  | **Control** | **Treated** | **Control** | **Treated** | **Control** | **Treated** | **Control** | **Treated** |
| **1.**  Li, 2016  China | 200mg/kg NaF solution | Every 10 mins till maturation of silkworm. | Every 10 mins till maturation of silkworm. | Intestinal Content | 5040  5737 | 5207  6136 | 11,403.44  12,373.26 | 11,665.57  14,024.97 | N/A | N/A | 5.53  5.99 | 5.62  6.33 |
| **2.**  Liu, 2019  China | 110.5 mg NaF, =50 mg F ion/L  221 mg NaF, =100 mg F ion/L | 70 days | 70 days | Cecal Contents | 566 | 620 | 616.1 | 684 | 0.165 | 0.065 | 3.108 | 4.045 |
| **3.**  Miao,2020  China | 400 (low-F) mg/kg F  1200 mg/kg F (high-F) | 16 hours | 16 hours | Small intestine and cecal contents | 2959 | N/A | N/A | N/A | Increased in high fluoride group | Increased in high fluoride group | N/A | N/A |
| **4.**  Xin,2021  China | 100 mg/l NaF  100mg/l NaF and L. johnsoniiBS15 probiotic | 98 days | 98 days | Kidney and colon | 20 | 86  18 | N/A | N/A | N/A | N/A | N/A | Increased |
| **5.**  Yan,2021  China | 100 mg/L NaF (F group) | 121 days | 121 days | Faeces | 456 | 542 | N/A | N/A | N/A | N/A | N/A | Increased |
| **6.**  Li,2021  China | 750mg/kg NaF in feed | 28 days | 28 days | Intestinal tissue | 300 | 97 | 401.13 ± 34.04 | 273.40 ± 81.53 | 5.47 ± 0.52 | 4.51 ± 1.71 | 0.91 ± 0.06 | 0.83 ± 0.19 |
| **7.**  Qiu,2020  United States | 100 mg/L NaF | 121 days | 121 days (parents were given fluoride for 21 days and then offspring until postnatal day 90.) | Faeces | 456 | 542 | 193.32 ± 7.34 | 152.80 ± 20.81 | N/A | N/A | 6.02 ± 0.31 | 6.25 ± 0.14 |
| **8.**  Zhang, 2022  China | 80 mg/L NaF | Assessed after 30,60 and 90 days | 90 days | Faeces | N/A | N/A | N/A | Decreased  Compared to control | N/A | N/A | N/A | Decreased  Compared to control |
| **9.**  Zhong, 2022  China | 50 mg/L sodium fluoride (NaF)  100 mg/L sodium fluoride (NaF)  150 mg/L sodium fluoride (NaF) | 12 weeks | 12 weeks | Faeces | N/A | N/A | 378.73±12.30 | 150 mg/L sodium fluoride (NaF)  368.71±10.42 | 0.94±0.03 | 0.93±0.04 0.88±0.05 0.93±0.03 | 4.00±0.29 | 3.92±0.50 3.61±0.37 3.86±0.26 |
| **10.**  Zhu, 2022  China | 25 mg/L F  50 mg/L F  100 mg/L F | 70 days | 70 days | Colonic content | 6003 | N/A | N/A | N/A | N/A | N/A | N/A | Decreased  Compared to control |
| **12.**  Zhang, 2023  China | 100 mg/L NaF  Antibiotics cocktail 10 mg/ml  F+ antibiotic  F + bacteria from faecal microbiota transplantation  SCFA group  F+ SCFA | 90 days | 90 days | Faeces | N/A | N/A | decreased | decreased | decreased | decreased | decreased | decreased |
| **11.**  Zhou, 2023  China | 100 mg/L NaF | 90 days | 90 days | Faeces | N/A | N/A | 535.71±65.49 | 491.42±50.70 | 0.90±0.033 | 0.90±0.032 | 4.78±0.53 | 4.54±0.24 |
| **12.**  Zhong, 2022  China | 25 mg/L sodium fluoride (NaF)  50 mg/L sodium fluoride (NaF)  100 mg/L sodium fluoride (NaF) | 12 weeks | 12 weeks | Faeces | N/A | N/A | 378.73±12.30 | 403.88±12.80 401.36±21.55 381.06±12.08 | 0.94±0.03 | 0.96±0.02 | 4.00±0.29 | 4.26±0.22 |
| **13.** Dashuan, 2023  China | 15 mg/kg F (15 mg/l F)  45 mg/kg F (45 mg/l F)  75 mg/kg F (75 mg/l F) | 3 months | 3 months | colon | 2240 | 15 mg/kg F (15 mg/l F)  1032  45 mg/kg F (45 mg/l F)  933  75 mg/kg F (75 mg/l F)  1293 | 1200 | 15 mg/kg F (15 mg/l F)  1000  45 mg/kg F (45 mg/l F)  800  75 mg/kg F (75 mg/l F)  800 | N/A | N/A | 7.9 | 15 mg/kg F (15 mg/l F)  7.7  45 mg/kg F (45 mg/l F)  7.7  75 mg/kg F (75 mg/l F)  7.7 |
| **14**. Guijie Chen, 2023  China | 50 mg/l NaF | 10 weeks | 10 weeks | colon | 440 | 450 | 450 | 460 | 1 | 1 | 6 | 6 |
| **15**. ZHANG Xiao Li, 2023  China | 100 mg/l NaF | 121 days | 121 days | colon | 2,026 | 2,208 | 2000 | 3000 | 1 | 1 | 7 | 7 |
| **16.** Huang, 2023 China | 24 mg/kg NaF ≡ 24 mg/L NaF | 8 weeks | 8 weeks | colon | N/A | N/A | No difference between the groups | No difference | No difference | No difference | No difference | No difference |
| **Human Studies** | | | | | | | | | | | | |
| **1.**  Zhou, 2023  China | N/A | N/A | N/A | Faeces | 155 | 318 | 681.87±139.09 | 653.49±153.10 | 0.95±0.03 | 0.94±0.03 | 6.03±0.53 | 5.88±0.51 |
| **2.** [Hai-Jeon Yoon](javascript:;), 2019  Korea | Fluorine-18-fluorodeoxyglucose (18F-FDG) intestinal uptake no dose mentioned | N/A | N/A | Faeces | N/A | Higher uptake  1160  Lower uptake  1092 | N/A | Higher uptake  0.8  Lower uptake  0.8 | N/A | Higher uptake  Lower uptake | N/A | Higher uptake  5.46 ± 0.71  Lower uptake  5.46 ± 0.71 |

**Supplementary Table 6 (S6): Effect of Fluoride dose and duration on microbiota-associated functions (gene expression and metabolites)**

| **Study ID** | **Dose of Fluoride** | **Duration of Exposure** | **Gene Expression** | | **Metabolites** | |
| --- | --- | --- | --- | --- | --- | --- |
|  |  |  | **Control Group** | **Intervention**  **Group** | **Control Group** | **Intervention Group** |
| **2.**  Davis,2012  Australia | 20mM fluoroacetate (added in media) | 72 hrs | N/A | N/A | Glycerol  lactate  ethylene glycol  citrate  ethanol | Glycerol  lactate  ethylene glycol  citrate  ethanol  **^#^(NS)** |
| **3.**  Dionizio, 2021  Australia | 10 mg/l F  50 mg/l F | 30 days | N/A | N/A | N/A | **(10 mg/l F)**  276 Proteins found.  Facilitated glucose transporter member 4.  Mitogen-activated protein kinase 3.  50-AMP-activated protein kinase catalytic subunit alpha-1.  50-AMP-activated protein kinase subunit beta-1  Polyubiquitin-6.  Dystrophin.  Myosin.  **(50 mg/l F)**  285 Proteins found  AMPK subunit alpha 1.  AMPK subunit beta 1.  Tumour necrosis factor.  Phosphoglycerate mutase 2.  Calcium-activated potassium channel subunit alpha-1.  Dynein light chain 1, cytoplasmic  Polyubiquitin-6.  Dystrophin↓  Calcium/calmodulin-dependent protein kinase kinase 1↓  Regulating synaptic membrane exocytosis protein 1.  Myosin |
| **4.**  Fu, 2019  China | 100 mg/l NaF | 60 days | N/A | N/A | N/A | GSH activity↓  SOD activity↓  CAT↓ |
| **5.**  Fu, 2022  China | 100 mg/l NaF | 6 months | N/A | N/A | IL-1β  IL-6  TNF-α  TLR2  NF-κB  Occludin  ZO-1  Claudin1  α-defensin5  Reg3b  Reg3g | IL-1β↑  IL-6 ↑  TNF-α↑  TLR2 ↑  NF-κB↑  Occludin ↓  ZO-1 ↓  Claudin1 ↓  α-defensin5↓  Reg3b↓  Reg3g↓ |
| **6.**  Komuroglu, 2021  Turkey | 100 ppm NaF | 12 weeks | N/A | N/A | MDA  SOD  catalase | MDA↑  SOD ↓  catalase ↓ |
| **7.**  Li, 2020  China | 4.76 mM NaF | As the study is an in vitro study so , it has taken place at different time points and duration depending upon the  tests to be performed | manXYZ  fruAB  gatABC  celB  ulaAB  bglF  orf  orf0939  malX(orf2148)  crr(orf2335)  Gls24 (orf2103)  cold shock protein (orf0572)  heat shock protein (orf0229)  heat-inducible transcription repressor HrcA(orf0228) thioredoxin (orf0326) | 237 DEGs  92 genes ↑  145 genes↓  GO database  113↑  181↓  manXYZ↓  fruAB↓  gatABC↓  celB↓  ulaAB↓  bglF↓  orf↓  orf0939↑  malX(orf2148)↑  crr(orf2335)↑  Gls24 (orf2103)↓  cold shock protein (orf0572)↓  heat shock protein (orf0229)↓  heat-inducible transcription repressor HrcA(orf0228) ↓ thioredoxin (orf0326)↓ | N/A | casease  lipase  amylase |
| **8.**  Li, 2016  China | 200mg/kg NaF solution | Every 10 mins from the fourth  instar to the third day of fifth instar. | N/A | N/A | Acetic acid  Propionic acid  Butyric acid  Isobutyric acid  Isovaleric acid | Isobutyric acid **^#^(NS)**  Isovaleric acid **^#^(NS)**  Acetic acid ↑  Propionic acid ↑  Butyric acid ↑ |
| **9.**  Li,2022  China | 200mg/kg NaF solution | 36 hours | Att2  CecA  Lys  CecB6  CecD  Leb1 | Att2↑  CecA↑  Lys↑  CecB6↓  CecD↓  Leb1↓ | N/A | arginine↑  glutamine↑  adenosine ↓  guanosine↓  Pyrimidine metabolism↑  purine metabolism↑  arginine biosynthesis↑  mineral absorption↓  protein digestion and absorption↓  aminoacyl-tRNA biosynthesis↓ |
| **10.**  Liu, 2019  China | 110.5 mg NaF, =50 mg F ion/L  221 mg NaF, =100 mg F ion/L | 70 days | N/A | N/A | glycoproteins | glycoproteins ↓ |
| **11.**  Liu, 2021  China | 100 mg/L NaF | 69 days | Beclin1  LC3  p62 | Beclin1↑  LC3↑  p62↓ | FSH  LH  testosterone | FSH **^#^(NS)**  LC3-II/LC3-I↑  p62↓  Beclin1↑ |
| **12.**  Miao, 2020  China | 400 (low-F) mg/kg F  1200 mg/kg F (high-F) | 16 hours | N/A | N/A | D-lactate  DAO  L-1β  IL-6  TNF-α  ZO-1  claudin-1  claudin-4  acetic acid  propionic acid  butyric acid  iso-pentanoic acid  isobutyric acid  pentanoic acid | **400 (low-F) mg/kg F**  D-lactate  DAO  L-1β  IL-6  TNF-α  acetic acid  propionic acid  butyric acid  iso-pentanoic acid  isobutyric acid  pentanoic acid **^#^(NS)**  **1200 mg/kg F (high-F)**  D-lactate ↑  DAO↑  L-1β↑  IL-6↑  TNF-α↑  ZO-2 ↓  claudin-4↓  ZO-1↓  claudin-1↓  claudin-4↓  acetic acid ↓  propionic acid↑  butyric acid ↓  iso-pentanoic acid ↓  isobutyric acid ↓  pentanoic acid |
| **13.**  Miao, 2020  China | 400 (low-F) mg/kg F  1200 mg/kg F (high-F) | 59 days | sIgA mRNA expression  MUC2 mRNA expression | sIgA mRNA expression↓  MUC2 mRNA expression ↓ | amylase  maltase  lactase  lipase  trypsin  Sucrase | **1200 mg/kg F (high-F)**  amylase↓  maltase↓  lactase↓  lipase  trypsin  Sucrase **^#^(NS)** |
| **14.**  Parthasaradhi, 2020  India | 10.0 mMNaF  20.0 mM NaF  30.0 mM NaF  40.0 mMNaF  50.0 mMNaF | 24 hrs | N/A | N/A | enolase enzyme | enolase enzyme↓ |
| **15.**  Sun,2020  China | 100 mg/l NaF  100mg/l NaF and L. johnsoniiBS15 probiotic | 70 days | Dbn  MAP-2  NMDAR  SYP | Dbn↓  MAP-2↓  NMDAR↓  SYP↓ | T-AOC  GSH-Px  SOD  MDA  amylase  trypsin  lipase | **100 mg/l NaF**  T-AOC↓  GSH-Px ↓  SOD  MDA↑  amylase↓  trypsin↓  lipase↓  sIgA↑  **100mg/l NaF and L. johnsoniiBS15 probiotic**  T-AOC **^#^(NS)**  GSH-Px **^#^(NS)**  SOD↑  MDA↓  amylase  trypsin  lipase **^#^(NS)** |
| **16.**  Wang, 2020  China | 100mg/l F  50mg/l F | 70 days | N/A | N/A | glycoproteins | **100 mg/l NaF**  glycoproteins ↓29.13%  **50 mg/l F**  glycoproteins↓ 13.20% |
| **17.**  Wang, 2019  China | 0.5 mg/L NaF  5 mg/L NaF  50 mg/L NaF | 105 days | N/A | N/A | N/A | Energy metabolic pathways downregulated. |
| **18.**  Xin, 2021  China | 100 mg/l NaF  100mg/l NaF and L. johnsoniiBS15 probiotic | 98 days | Occludin  ZO-1  Claudin-1 | Occludin↓  ZO-1↓  Claudin-1↑ | N-acetyl-beta-D-glucosaminidase (NAG) | 100 mg/l NaF  N-acetyl-beta-D-glucosaminidase (NAG)↓ |
| **19.**  Xin, 2021  China | 100 ppm NaF ≈ 37.8 ±2.4 ppm F ̅  L. johnsonii BS15 (prob group; 0.2 mL/day) | 70 days | N/A | N/A | Brain-Derived Neurotrophic Factor (BDNF)  CREB, cAMP response element-binding protein  NCAM  SCF mRNA  PLP  MOG  MBP  MAG  Bcl-2  Bcl- xl  Bax  Bad  caspase3 (highest)  caspase9  ZO-1  claudin-1  occludin  D-lactate (lower) | **100 ppm NaF ≈ 37.8 ±2.4 ppm F ̅**  Brain-Derived Neurotrophic Factor (BDNF)↓  CREB, cAMP response element-binding protein↓  NCAM  SCF mRNA↓  PLP↓  MOG  MBP  MAG↓  Bcl-2  Bcl- xl ↓  Bax ↑  Bad ↑  caspase3  caspase9 (highest)  ZO-1 ↓  claudin-1↓  occludin ↓  D-lactate↑  **L. johnsonii BS15 (prob group; 0.2 mL/day)**  Brain-Derived Neurotrophic Factor (BDNF)  CREB, cAMP response element-binding protein  NCAM  SCF mRNA (slightly higher)  PLP  MOG  MBP  MAG (reduced compared to control)  Bcl-2  Bcl- xl  Bax  Bad  caspase3  caspase9  ZO-1  claudin-1↓  occludin ↓ |
| **20.**  Yasuda, 2017  USA | 4ppm F in drinking water  4ppm F + 2.25ug of fluoride per day via gavage | 12 weeks | N/A | N/A | N/A | **4ppm F**  glyoxylate cycle↓  succinate dehydrogenase↓  mevalonate (MVA)↓  **4ppm F + 2.25ug of fluoride per day via gavage**  glyoxylate cycle↓  succinate dehydrogenase↓  mevalonate (MVA)↓ |
| **21.**  Yu, 2020  China | 80 mg/L NaF | 90 days | N/A | N/A | ZO-1  occludin  LPS | ZO-1↓  occludin↓  LPS↑ |
| **22.**  Zhang, 2022  China | 80 mg/L NaF | 90 days | IL-6  IL-1β  TNF-α  IL-10  TGF-β | 30 days exposure  IL-6↑  IL-1β↑  TNF-α↑  IL-10 ↑  TGF-β↑  60 days exposure  IL-6↑  IL-1β↑  TNF-α↑  IL-10 ↑  TGF-β↑  90 days exposure  IL-6 ↓  IL-1β ↓  TNF-α ↓  IL-10 ↓  TGF-β ↓ | MDA  SOD  CAT  GSH level  GPx  ACP  LZM  Muc2  ZO-1  Occludin  Claudin-1 | **80 mg/L of F as sodium fluoride**  **30 days exposure**  MDA (96.3%) ↑  SOD (30.4%) ↑  CAT (40.7%) ↑  GSH level (50.6%) ↓  GPx (84.4%) ↓  ACP (15.9%) ↓  LZM (25.9%) ↑  Muc2 ↓  ZO-1 **^#^(NS)**  **60 days exposure**  ROS↑  MDA↑  AKP ↑  MPO↑  Muc2 ↓  ZO-1↓  Occludin↓  Claudin-1↑  **90 days exposure**  SOD↑  CAT↓  GSH↓  GPx↓  ACP↓  LZM↓  Muc2 ↓  ZO-1↓  Occludin↓  Claudin-1↓ |
| **23.**  Zhu, 2022  China | 25 mg/L F  50 mg/L F  100 mg/L F | 70 days | IL-17A  IL-22  IL-17RA | **25 mg/L F**  IL-17A↑  IL-22↑  IL-22R  IL-17RA ↓  **50 mg/L F**  IL-17A↑  IL-22↑  IL-22R↑  IL-17RA ↓  **100 mg/L F**  IL-17A↑  IL-22↑  IL-22R↑  IL-17RA ↓ | glycoproteins | **25 mg/L F**  -glycoproteins  -CRAMP↑↑  -β-Defensin-1↑  -β-Defensin-3↑  **50 mg/L F**  glycoproteins↓  -CRAMP↑  -β-Defensin-1↑  -β-Defensin-3↑  **100 mg/L F**  glycoproteins↓  -CRAMP↑  -β-Defensin-1↑  -β-Defensin-3↑  Gene expression  **25 mg/L F**  IL-17A↑  IL-22↑  IL-22R  IL-17RA ↓  **50 mg/L F**  IL-17A↑  IL-22↑  IL-22R↑  IL-17RA ↓  **100 mg/L F**  IL-17A↑  IL-22↑  IL-22R↑  IL-17RA ↓ |
| **24.**  Zhang, 2023  China | 100 mg/L NaF  Antibiotics cocktail 10 mg/ml  F+ antibiotic  F + bacteria from faecal microbiota transplantation  SCFA group  F+ SCFA | 90 days | [TLR2](https://www.sciencedirect.com/topics/pharmacology-toxicology-and-pharmaceutical-science/toll-like-receptor-2)  [Myd88](https://www.sciencedirect.com/topics/pharmacology-toxicology-and-pharmaceutical-science/myeloid-differentiation-factor-88)  [TRAF6](https://www.sciencedirect.com/topics/pharmacology-toxicology-and-pharmaceutical-science/tumor-necrosis-factor-receptor-associated-factor-6)  IKKβ  TNF-α  IL-1β  IL-6  IFN-γ  IL-10  TGF-β | TNF-α↑  IL-1β↑  IL-6↑  IFN-γ ↑  IL-10 ↑  TGF-β ↑  [TLR2](https://www.sciencedirect.com/topics/pharmacology-toxicology-and-pharmaceutical-science/toll-like-receptor-2) ↑  [Myd88](https://www.sciencedirect.com/topics/pharmacology-toxicology-and-pharmaceutical-science/myeloid-differentiation-factor-88) ↑  [TRAF6](https://www.sciencedirect.com/topics/pharmacology-toxicology-and-pharmaceutical-science/tumor-necrosis-factor-receptor-associated-factor-6) ↑  IKKβ ↑ | [TLR2](https://www.sciencedirect.com/topics/pharmacology-toxicology-and-pharmaceutical-science/toll-like-receptor-2)  [Myd88](https://www.sciencedirect.com/topics/pharmacology-toxicology-and-pharmaceutical-science/myeloid-differentiation-factor-88)  [TRAF6](https://www.sciencedirect.com/topics/pharmacology-toxicology-and-pharmaceutical-science/tumor-necrosis-factor-receptor-associated-factor-6)  IKKβ  TNF-α  IL-1β  IL-6  IFN-γ  IL-10  TGF-β  Acetate  Propionate  Butyrate | **100 mg/l NaF**  TNF-α↑  IL-1β↑  IL-6↑  IFN-γ ↑  IL-10 ↑  TGF-β ↑  [TLR2](https://www.sciencedirect.com/topics/pharmacology-toxicology-and-pharmaceutical-science/toll-like-receptor-2) ↑  [Myd88](https://www.sciencedirect.com/topics/pharmacology-toxicology-and-pharmaceutical-science/myeloid-differentiation-factor-88) ↑  [TRAF6](https://www.sciencedirect.com/topics/pharmacology-toxicology-and-pharmaceutical-science/tumor-necrosis-factor-receptor-associated-factor-6) ↑  IKKβ ↑  [TLR](https://www.sciencedirect.com/topics/pharmacology-toxicology-and-pharmaceutical-science/toll-like-receptor-2)4 ↑  Acetate ↓  Propionate↓  Butyrate↓ |
| **25.**  Zhou, 2023  China | 100 mg/L NaF | 90 days | N/A | N/A | N/A | Pentose  Glucuronate  α-ketoglutaric acid ↑ |
| **26**  Tian, 2023  China | 100 mg/L NaF | 120 days | N/A | N/A | Glutamine  alpha ketoglutarate | Creatinine ↑  Beclin1↑  Glutamine↓  alpha ketoglutarate↓ |
| **27.** Huang, 2023 China | 24 mg/kg NaF ≡ 24 mg/L NaF |  | SLC7A11  TBARS  GPX4  PTGS2  CHAC1  Rgs4 | SLC7A11↑  TBARS↑  GPX4↓  PTGS2↑  CHAC1↑  Rgs4 ↓ | glutathione | glutathione↓ |
| **28.** ZHANG Xiao Li, 2023  China | 100 mg/l NaF | 8 weeks | N/A | N/A | linoleic acid metabolism, tryptophan metabolism, lipoic acid metabolism, and α-linolenic acid metabolism | linoleic acid metabolism, tryptophan metabolism, lipoic acid metabolism, and α-linolenic acid metabolism ↑ |
| **29.** Dashuan, 2023  China | 15 mg/kg F (15 mg/l F)  45 mg/kg F (45 mg/l F)  75 mg/kg F (75 mg/l F) | 18 weeks | MDA  SOD  GSH  NQO1  Nrf2  HQ1 | MDA ↓  SOD ↑  GSH↑  NQO1↓  Nrf2 ↓  HQ1 ↓ | MDA  SOD  GSH  NQO1  Nrf2  HQ1 | MDA ↓  SOD ↑  GSH↑  NQO1↓  Nrf2 ↓  HQ1 ↓ |
| **30.** Guijie Chen, 2023  China | 50 mg/l NaF | 6 weeks | ZO-1  occludin  IL-1β, IL-6, TNF-α  Myd88  Tlr4 | ZO-1↓  occludin↓  IL-1β ↑, IL-6↑, TNF-α↑  Myd88↑  Tlr4↑ | ZO-1  occludin  IL-1β, IL-6, TNF-α  Myd88  Tlr4 | ZO-1↓  occludin↓  IL-1β ↑, IL-6↑, TNF-α↑  Myd88↑  Tlr4↑ |
| **31**. Taotao Zhao, 2024  China | 100 mg/l NaF | 121 days | BDNF-PI3K/AKT  PI3K  Bak  Bax  Caspase-7  protein Caspase-3 | BDNF-PI3K/AKT↓  PI3K↑  Bak↑  Bax↑  Caspase-7↑  protein Caspase-3↑ | BDNF-PI3K/AKT  PI3K  Bak  Bax  Caspase-7  protein Caspase-3 | BDNF-PI3K/AKT↓  PI3K↑  Bak↑  Bax↑  Caspase-7↑  protein Caspase-3↑ |
| **32**. Zhe Mo,2023  China | 25 mg/l NaF  50 mg/l NaF  100 mg/l NaF  150 mg/l NaF | 3 months | N/A | N/A | D-lyxose ketol-isomerase,  alanine-synthesizing transaminase,  Fis family transcriptional regulator,  CoA-dependent NAD(P)H sulfur oxidoreductase,  3D-(3,5/4)-trihydroxycyclohexane-1,2-dione acylhydro-lase (decyclizing) | 25 mg/l NaF  D-lyxose ketol-isomerase,↑  alanine-synthesizing transaminase, ↑  Fis family transcriptional regulator, ↑  CoA-dependent NAD(P)H sulfur oxidoreductase, ↑  3D-(3,5/4)-trihydroxycyclohexane-1,2-dione acylhydro-lase (decyclizing)↑  50 mg/l NaF  D-lyxose ketol-isomerase,↑  alanine-synthesizing transaminase, ↑  Fis family transcriptional regulator, ↑  CoA-dependent NAD(P)H sulfur oxidoreductase, ↑  3D-(3,5/4)-trihydroxycyclohexane-1,2-dione acylhydro-lase (decyclizing)↑  100 mg/l NaF  D-lyxose ketol-isomerase,↑  alanine-synthesizing transaminase, ↑  Fis family transcriptional regulator, ↑  CoA-dependent NAD(P)H sulfur oxidoreductase, ↑  3D-(3,5/4)-trihydroxycyclohexane-1,2-dione acylhydro-lase (decyclizing)↑  150 mg/l NaF  D-lyxose ketol-isomerase,↑  alanine-synthesizing transaminase, ↑  Fis family transcriptional regulator, ↑  CoA-dependent NAD(P)H sulfur oxidoreductase, ↑  3D-(3,5/4)-trihydroxycyclohexane-1,2-dione acylhydro-lase (decyclizing)↑ |
| **33.** Wu, Yue, 2024  China | 100 mg/l NaF | 10 weeks | IL-1β, IL-6, TNF-α  ASBT, IBABP, OST-α, and OST-β | IL-1β ↑, IL-6↑, TNF-α↑ | IL-1β, IL-6, TNF-α  ASBT, IBABP, OST-α, and OST-β | IL-1β ↑, IL-6↑, TNF-α↑ |
| **34.** Chenjun Zhao, 2024  China | 200 mg/l NaF | 60 days | IL-1β, IL-6, TNF-α  [Occludin](https://www.sciencedirect.com/topics/pharmacology-toxicology-and-pharmaceutical-science/occludin)  mucin-2 mRNA | IL-1β ↑, IL-6↑, TNF-α↑  [Occludin](https://www.sciencedirect.com/topics/pharmacology-toxicology-and-pharmaceutical-science/occludin)↑  mucin-2 mRNA↑  GPRCs↓ | IL-1β, IL-6, TNF-α  [Occludin](https://www.sciencedirect.com/topics/pharmacology-toxicology-and-pharmaceutical-science/occludin)  mucin-2 mRNA | IL-1β ↑, IL-6↑, TNF-α↑  [Occludin](https://www.sciencedirect.com/topics/pharmacology-toxicology-and-pharmaceutical-science/occludin)↑  mucin-2 mRNA↑  GPRCs↓ |
| **35**. Yueying Feng, 2024  China | 0.57 mg/L F35  5.7 mg/L F35 | 12 weeks | IL-1β  TNF-α  IL-10  [TLR4](https://www.sciencedirect.com/topics/medicine-and-dentistry/toll-like-receptor-4),  NF-κB  ZO-1  occludin | 0.57 mg/L F35  IL-1β  TNF-α  IL-10↓  [TLR4](https://www.sciencedirect.com/topics/medicine-and-dentistry/toll-like-receptor-4), ↑  NF-κB↑  5.7 mg/L F35  IL-1β↑  TNF-α↑  IL-10↓  [TLR4](https://www.sciencedirect.com/topics/medicine-and-dentistry/toll-like-receptor-4), ↑  NF-κB↑  SOD↓  CAT↓  GSH↓  MDA↑  AKT↓  PI3K↓  ZO-1↓  occludin↓ | IL-1β  TNF-α  IL-10  [TLR4](https://www.sciencedirect.com/topics/medicine-and-dentistry/toll-like-receptor-4),  NF-κB  ZO-1  occludin | 0.57 mg/L F35  IL-1β  TNF-α  IL-10↓  [TLR4](https://www.sciencedirect.com/topics/medicine-and-dentistry/toll-like-receptor-4), ↑  NF-κB↑  5.7 mg/L F35  IL-1β↑  TNF-α↑  IL-10↓  [TLR4](https://www.sciencedirect.com/topics/medicine-and-dentistry/toll-like-receptor-4), ↑  NF-κB↑  SOD↓  CAT↓  GSH↓  MDA↑  AKT↓  PI3K↓  ZO-1↓  occludin↓ |
|  |  | 5 weeks | **Human Studies** |  |  |  |
| **Study ID** | **Dose of fluoride** | Postnatal 21 days | **Gene expression** | | **Metabolites** | |
|  |  |  | **Control Group** | **Intervention**  **Group** | **Control Group** | **Intervention**  **Group** |
| **1.**  Chen, 2021  China | 1 mg/l F  2 mg/l F  10 mg/l F  15 mg/l F | 24 Hrs | N/A | N/A | Acetic acid  propionic acid  butyric acid | Acetic acid  propionic acid  butyric acid **^#^(NS)**  At 1 and 2 mg/l F no effect on KEGG pathway, but higher concentrations changed the functional modules. |
| **2.**  Zhou, 2023  China | N/A | N/A | N/A | N/A | N/A | Pentose  Glucuronate |
| **3.**  Wang, 2023  China | N/A | N/A | N/A | N/A | 5-hydroxyindoleacetic  acid Tryptamine  Indole acetaldehyde | 5-hydroxyindoleacetic  acid ↓  Tryptamine ↓  Indole acetaldehyde ↓ |

*** Control= Without Fluoride exposure**; *** Intervention Group = Those which are given fluoride as a treatment/intervention**; Abbreviations: ↓, decrease; ↑, increase; N/A not applicable/not mentioned; LC/MS, liquid chromatography mass spectrometry; parts per million; SCFA, short chain fatty acid; mg/l milligram per litre; cyt, cytochrome; GSH, glutathione; SOD, [Superoxide dismutase; CAT, catalase; MDA, Malondialdehyde; FSH, follicle stimulating hormone; LC3, Microtubule-associated protein light chain 3; p62, Ubiquitin-binding protein; DAO, diamine oxidase; GPX Glutathione peroxidase; ACP, acid phosphatase; LZM, lysozyme; IL6, cytokine; TNF α, tumour necrosis factor; ZO1, zonula occludens; CREB, cAMP response elements binding protein; NCAM, neural cell adhesion molecule; PLP pyridoxal phosphate; MOG,](https://en.wikipedia.org/wiki/Superoxide_dismutase) [Myelin Oligodendrocyte Glycoprotein; MBP, Myelin basic protein; BCL, B-cell lymphoma; Bax, BCL2‑associated X protein; AKP, alkaline phosphatase; CRAMP, Cathelicidin-related anti-microbial peptide; TGF, transforming growth factors; TLR, toll- like receptors; TRAF, Tumor necrosis factor receptor–associated factor; IKK β, inhibitor of nuclear factor kappa-B kinase subunit beta.](https://www.ncbi.nlm.nih.gov/pmc/articles/PMC7795410/)

**^[#](https://en.wikipedia.org/wiki/Superoxide_dismutase)^[(NS)= non-significant difference between the two groups](https://en.wikipedia.org/wiki/Superoxide_dismutase)**
